# Satellite DNA-Like Elements Associated With Genes Within Euchromatin of the Beetle *Tribolium castaneum*

**DOI:** 10.1534/g3.112.003467

**Published:** 2012-08-01

**Authors:** Josip Brajković, Isidoro Feliciello, Branka Bruvo-Mađarić, Đurđica Ugarković

**Affiliations:** * Department of Molecular Biology, Ruđer Bošković Institute, Bijenička 54, HR-10000 Zagreb, Croatia, and; † Dipartimento di Medicina Clinica e Sperimentale, Università degli Studi di Napoli Federico II, via Pansini 5, I80131, Napoli, Italy

**Keywords:** repetitive DNA, satellite DNA, gene regulation, transposon, immunoglobulin-like genes

## Abstract

In the red flour beetle *Tribolium castaneum* the major TCAST satellite DNA accounts for 35% of the genome and encompasses the pericentromeric regions of all chromosomes. Because of the presence of transcriptional regulatory elements and transcriptional activity in these sequences, TCAST satellite DNAs also have been proposed to be modulators of gene expression within euchromatin. Here, we analyze the distribution of TCAST homologous repeats in *T. castaneum* euchromatin and study their association with genes as well as their potential gene regulatory role. We identified 68 arrays composed of TCAST-like elements distributed on all chromosomes. Based on sequence characteristics the arrays were composed of two types of TCAST-like elements. The first type consists of TCAST satellite-like elements in the form of partial monomers or tandemly arranged monomers, up to tetramers, whereas the second type consists of TCAST-like elements embedded with a complex unit that resembles a DNA transposon. TCAST-like elements were also found in the 5′ untranslated region (UTR) of the CR1-3_TCa retrotransposon, and therefore retrotransposition may have contributed to their dispersion throughout the genome. No significant difference in the homogenization of dispersed TCAST-like elements was found either at the level of local arrays or chromosomes nor among different chromosomes. Of 68 TCAST-like elements, 29 were located within introns, with the remaining elements flanked by genes within a 262 to 404,270 nt range. TCAST-like elements are statistically overrepresented near genes with immunoglobulin-like domains attesting to their nonrandom distribution and a possible gene regulatory role.

Based on the hypothesis of Britten and Davidson (1971), repetitive elements can be a source of regulatory sequences and act to distribute regulatory elements throughout the genome. In particular, mobile transposable elements (TEs) are predicted to be a source of noncoding material that allows for the emergence of genetic novelty and influences evolution of gene regulatory networks (Feschotte 2008). Recently it has been shown that at least 5.5% of conserved noncoding elements unique to mammals originate from mobile elements and are preferentially located close to genes involved in development and transcription regulation (Lowe *et al.* 2007). The complete sequence conservation, wide evolutionary distribution, and presence of functional elements such as promoters and transcription factor binding sites within some satellite DNA sequences has led to the assumption that in addition to participating in centromere formation, they might also act as *cis*-regulatory elements of gene expression (Ugarković 2005). To perform potential regulatory functions, satellite DNA elements are predicted to be preferentially distributed in euchromatic portion of the genomes in the vicinity of genes. Whole-genome sequencing projects enable the presence and distribution of satellite DNA repeats in the euchromatic portion of the genome to be determined. The analysis of satellite DNA-like elements dispersed within euchromatin, and their comparison with homologous elements present within heterochromatin, also may reveal insights into the origin of satellite DNAs and their subsequent evolution (Kuhn *et al.* 2012).

Satellite DNAs are major building elements of pericentromeric and centromeric heterochromatin in many eukaryotic species, and in certain species they account for the majority of genomic DNA, as in beetles from the coleopteran family Tenebrionidae (Ugarković and Plohl 2002). In the red flour beetle *Tribolium castaneum*, pericentromeric heterochromatin comprises approximately 40% of the genome, and TCAST satellite DNA has previously been characterized as the major satellite that encompasses centromeric as well as pericentromeric regions of all 20 chromosomes (Ugarković *et al.* 1996). TCAST satellite is composed of two subfamilies, Tcast1a and Tcast1b, which together comprise 35% of the whole genome. Tcast1a and Tcast1b have an average homology of 79% and are a similar size at 362 bp and 377 bp, respectively, but they are characterized by a divergent, subfamily specific region of approximately 100 bp (Feliciello *et al.* 2011). The genome sequencing project of *T. castaneum* has recently been completed (Richards *et al.* 2008). Sequencing involved the euchromatic portion of the genome, with >20% of the genome, corresponding to heterochromatic regions, excluded due to technical difficulties.

In this article, we searched for the presence of TCAST satellite-homologous elements within the assembled *T. castaneum* genome by using a comprehensive computational analysis. By searching the sequenced *T. castaneum* genome, we found 68 TCAST satellite DNA arrays within the euchromatin of all chromosomes. They were mapped to 5′ or 3′ ends, as well as within introns, of more than 100 protein-coding genes. Based on sequence characteristics, dispersed TCAST-like elements were classified into two groups. The first group includes partial TCAST satellite monomers or short arrays of tandemly arranged monomers up to tetramers. The second group contains TCAST-like element embedded within complex repeat units that contain two hallmarks of DNA transposons, terminal inverted repeats and target-size duplications. The evolutionary relationship and possible modes of dispersion of the two types of dispersed TCAST-like sequences are discussed. In addition, we examined the sequence divergence, phylogenetic relationship, and chromosomal distribution of the elements. Annotation, characterization, and classification of genes within the region of TCAST-like elements are reported, with the preferential localization of TCAST-like elements near specific groups of genes identified. Our results demonstrate for the first time, the enrichment of satellite DNA-like elements in the vicinity of genes with immunoglobulin-like domains and suggest their possible gene-regulatory role.

## Materials and Methods

BLASTN version 2.2.22+ was used to screen the NCBI refseq_genomic database of *T. castaneum*. All scaffolds that have not been mapped to linkage groups were also screened. The program was optimized to search for highly similar sequences (megablast) to the query sequence [TCAST consensus sequence (Ugarković *et al.* 1996)]. Genes flanking TCAST–homologous elements were found automatically by NCBI blast. Sequences corresponding to hits, as well as their flanking regions, were analyzed by dot plot (http://www.vivo.colostate.edu/molkit/dnadot/), using standard parameters (window size 9, mismatch limit 0), or more relaxed conditions (window size 11, mismatch limit 1), to determine the exact start and end site of specific TCAST-like elements. The TCAST transposon-like elements were analyzed in detail for the presence of hallmarks such as terminal inverted repeats (TIRs) and target-site duplications with the aid of the Gene Jockey sequence analysis program (for Apple Macintosh). Secondary structures were determined using the default parameters of the MFOLD program available online [http://mfold.rna.albany.edu/?q=mfold (Zuker 2003)]. AT content was analyzed using BioEdit Sequence Alignment Editor (Hall 1999). Repbase, a reference database of eukaryotic repetitive DNA, was screened using WU-BLAST (Kohany *et al.* 2006).

Sequence alignment was performed using MUSCLE algorithm (Edgar 2004) combined with manual adjustment. All sequences were included in the alignment, with the exception of the ones that did not at least partially overlap with other sequences. Gblocks was used to eliminate poorly aligned positions and divergent regions of the alignments (Talavera and Castresana 2007). Alignments (original fasta files) are available upon request. jModelTest 0.1.1 software (Posada 2008) was used to infer best-fit models of DNA evolution—TPM3uf+G for transposon-like and A type elements and TPM1uf for B type elements. Maximum likelihood (ML) trees were estimated with the PhyML 3.0 software (Guindon and Gascuel 2003) using best-fit models. Markov chain Monte Carlo Bayesian searches were performed in MrBayes v. 3.1.2. (Huelsenbeck and Ronquist 2001) under the best-fit models (two simultaneous runs, each with four chains; 3 × 10^6^ generations; sampling frequency one in every 100 generations; majority rule consensus trees constructed based on trees sampled after burn-in). Branch support was evaluated by bootstrap analysis (1000 replicates) in ML and by posterior probabilities in Bayesian analyses. Pairwise sequence diversity (uncorrected *P*) was calculated using the MEGA 5.05 software (Tamura *et al.* 2011).

*T. castaneum* gene homologs in *Drosophila melanogaster* were searched using the OrthoDB Phylogenomic database. Each gene has OrthoDB identificator, with Uniprot data linked to OrthoDB (Waterhouse *et al.* 2011). To find sets of biological annotations that frequently appear together and are significantly enriched in a set of genes located near TCAST-like elements, program GeneCodis 2.0 available online (http://genecodis.dacya.ucm.es/) was used. GeneCodis generates statistical rank scores for single annotations and their combinations. To find all the possible combinations of annotations, GeneCodis uses the *apriori* algorithm introduced by Agrawal *et al.* 1993. Once the annotations were extracted, a statistical analysis based on the hypergeometric distribution or the χ^2^ test of independence was executed to calculate the statistical significance (*P* values) for each individual annotation or co-annotations.

Two-tailed hypergeometric test with Bonferroni correction (alpha = 0.025) was used to analyze the distribution of TCAST-like elements among *T. castaneum* chromosomes. In each chromosome the frequency of TCAST-like elements was compared with the frequency in the complete sample and the significance of deviations was calculated.

## Results

### Identification of dispersed TCAST-like elements

Using the consensus sequence of TCAST satellite DNA (Ugarković *et al.* 1996) as a query sequence, we screened the NCBI refseq_genomic database of *T. castaneum* with the alignment program BLASTN version 2.2.22+. The program was optimized to search for highly similar sequences (megablast) and blast hits on the query sequence were analyzed individually. Alignments were mapped regarding start and end site, chromosome number, and total length. When the distance between two alignments on the same chromosome was short, the genomic sequence was further analyzed by dot plot to identify any potential continuity between the two alignments. Only genomic sequences with at least 140 nt (40% of TCAST monomer length) of continuous sequence and >80% identity to the TCAST consensus sequence were considered for further analysis. The total number of dispersed TCAST-like elements was 68, with 36 elements flanked by genes at both 5′ and 3′ ends, 3 elements flanked by a single gene either at 5′ or 3′ end (sequences no. 36, 39, 50), and the 29 elements positioned within introns (Table 1). Except 68 TCAST-like elements associated with genes, no other dispersed TCAST-like elements were found within the assembled *T. castaneum* genome. Analysis of scaffolds that have not been mapped to linkage groups revealed the presence of an additional 41 TCAST-like elements, but because they were not mapped to *T. castaneum* genome and could possibly derive from heterochromatin, we did not consider them for further analysis.

**Table 1:** TCAST-like elements associated with genes within *T. castaneum* euchromatin

Uniprot	Entrez	Gene Name	Chr	Sat_seq.	Position	Distance, bp	DM Homolog	FBgn	Type	Length	Copies
D6WZP1	662564	Altered disjunction	9	1	5′	18,773	Q9VEH1	FBgn0000063	Satellite	734	2.0
D6WZP3	662624	Ras-related protein Rab-26	9	1	3′	7795	Q9VP48	FBgn0086913	Satellite	734	2.0
D6WZL9	661947	Probable serine/threonine-protein kinase	9	2	Inside		Q0KHT7	FBgn0052666	Satellite	993	2.8
D6X226	660275	Arrest	9	3	5′	99,669	Q8IP89	FBgn0000114	Satellite	716	2.0
D6X238	661741	Numb	9	3	3′	115,984	P16554	FBgn0002973	Satellite	716	2.0
	100141832	no match on uniprot	9	4	5′	1520			Satellite	517	1.4
D6X2D0	660440	Short-chain dehydrogenase	9	4	3′	6704	Q9VE80	FBgn0038610	Satellite	517	1.4
D6X1E7	656884	Cytochrome P450 306A1	9	5	5′	404,270	Q9VWR5	FBgn0004959	Satellite	1058	2.9
D6X2U7	656977	Elongase	9	5	3′	9947	Q9VCY6	FBgn0038986	Satellite	1058	2.9
D6X2C4	660195	Dopamine receptor 1	9	6	Inside		P41596	FBgn0011582	Satellite	304	0.8
D6X2U7	656977	Elongase	9	7	5′	7128	Q9VCY6	FBgn0038986	Satellite	394	1.1
D6X366	657055	elongation of very long chain fatty acids protein	9	7	3′	50,111	Q9VCY5	FBgn0053110	Satellite	394	1.1
D6X0D7	657748	Ret oncogene	9	8	5′	56,625	Q8INU0	FBgn0011829	Satellite	213	0.6
D6X0E1	657829	Dpr9	9	8	3′	62,781	Q9VFD9	FBgn0038282	Satellite	213	0.6
D6X2H8	654954	ADAM metalloprotease	9	9	Inside		Q6QU65	FBgn0051314	Transposon	1107	
D6X2U7	655561	Elongase	9	10	5′	47,902	Q9VCZ0	FBgn0038983	Transposon	1085	
D6X2V3	655640	Putative uncharacterized protein	9	10	3′	67,953	Q9VDB7	FBgn0038881	Transposon	1085	
D6X244	655011	Serine/threonine-protein kinase 32B	9	11	Inside		Q0KID3	FBgn0052944	Transposon	1062	
D6X374	100141521	Putative uncharacterized protein	9	12	Inside		Q9VGZ4	FBgn0037814	Satellite	292	0.8
D6X2C4	660195	Dopamine receptor 1	9	13	Inside		P41596	FBgn0011582	Transposon	900	
D6X259	656290	Transport and Golgi organization 13	9	14	5′	9456	Q9VGT8	FBgn0040256	Satellite	222	0.6
D6X260	656373	Protein-tyrosine sulfotransferase	9	14	3′	33,523	Q9VYB7	FBgn0086674	Satellite	222	0.6
D6X075	658603	MICAL-like protein	9	15	5′	39,684	Q9VU34	FBgn0036333	Satellite	203	0.6
D6X1P2	658891	tiptop	9	15	3′	142,821	Q9U3V5	FBgn0028979	Satellite	203	0.6
D6X095	659195	Troponin C	9	16	5′	3922	P47947	FBgn0013348	Transposon	589	
D6X0I1	659336	Troponin C	9	16	3′	15,143	P47947	FBgn0013348	Transposon	589	
D6X1J0	655713	Transporter	9	17	Inside		Q9NB97	FBgn0034136	Satellite	915	2.5
D6WF56	100141877	zinc finger protein 250	3	18	5′	125,685	Q7KAH0	FBgn0027339	Satellite	1208	3.4
D6WF61	656924	Transcription initiation factor TFIID subunit 7	3	18	3′	64,294	Q9VHY5	FBgn0024909	Satellite	1208	3.4
D6WGB1	659040	Mahya	3	19	5′	115,537	P20241	FBgn0002968	Satellite	687	1.9
D6WGB5	659201	V-type proton ATPase subunit E	3	19	3′	82,217	P54611	FBgn0015324	Satellite	687	1.9
D6WII0	100141571	NADH dehydrogenase, putative	3	20	5′	4278	Q9W3N7	FBgn0029971	Transposon	635	
D6WII2	100142263	Putative uncharacterized protein	3	20	3′	18,585	Q9VIY1	FBgn0032769	Transposon	635	
D6WFT8	657535	WD repeat-containing protein 47	3	21	Inside		Q960Y9	FBgn0026427	Satellite	604	1.7
D6WDY2	656125	Kynurenine aminotransferase	3	22	5′	10,696	Q8SXC2	FBgn0037955	Transposon	1000	
D6WDY4	656298	Annexin IX	3	22	3′	11,599	P22464	FBgn0000083	Transposon	1000	
D6WFK8	656174	ankyrin 2,3/unc44	3	23	Inside		Q7KU95	FBgn0085445	Transposon	1081	
D6WFX1	657874	ral guanine nucleotide exchange factor	3	24	5′	64,025	Q8MT78	FBgn0034158	Transposon	888	
D6WFX3	658031	galactose-1-phosphate uridylyltransferase	3	24	3′	3958	Q9VMA2	FBgn0031845	Transposon	888	
D6WDQ4	659233	Putative uncharacterized protein	3	25	5′	15,051	Q8T0R9	FBgn0038809	Transposon	1016	
D6WDQ6	659376	coiled-coil domain containing 96	3	25	3′	9162	A1ZA72	FBgn0013988	Transposon	1016	
D6WF68	655042	glucose dehydrogenase	3	26	5′	25,896	Q9VY00	FBgn0030598	Transposon	1067	
C3XZ92	655348	Mitogen-activated protein kinase kinase kinase kinase 2	3	26	3′	92,876	Q8SYA1	FBgn0034421	Transposon	1067	
D6WE82	658463	Putative uncharacterized protein	3	27	Inside		Q9VDK2	FBgn0038815	Transposon	314	
D6WHX6	658191	Putative uncharacterized protein	3	28	5′	173,881	Q1RKQ9	FBgn0085382	Transposon	826	
D6WI58	658343	Cathepsin L	3	28	3′	82,559	Q95029	FBgn0013770	Transposon	826	
D6WDJ9	656922	Putative uncharacterized protein	3	29	5′	173,548	Q8IPJ1	FBgn0031859	Transposon	1084	
D6WDN0	657559	PRMT5	3	29	3′	383,809	Q9U6Y9	FBgn0015925	Transposon	1084	
D6WGS3	656976	Putative uncharacterized protein	3	30	5′	37572	A0AMQ8	FBgn0034655	Satellite	216	0.6
D6WGT0	100142515	calpain 3	3	30	3′	226,707	Q11002	FBgn0008649	Satellite	216	0.6
D6WDS8	660532	Muscle-specific protein 300	3	31	5′	378,626	Q4ABG9	FBgn0260952	Transposon	1060	
D6WDT0	654860	Phosphatidylinositol-binding clathrin assembly protein	3	31	3′	7855	C1C3H4	FBgn0086372	Transposon	1060	
D6WHF2	664188	Nephrin	3	32	Inside		Q9W4T9	FBgn0028369	Transposon	666	
D6WI96	100142620	Heat shock protein 70	3	33	Inside		P11147	FBgn0001219	Transposon	1058	
D6WG02	654917	N-acetylglucosaminyltransferase vi	3	34	Inside		Q9VUH4	FBgn0036446	Transposon	319	
D6WYD1	656891	Putative uncharacterized protein	8	35	5′	385,712	Q8SY79	FBgn0032249	Satellite	625	1.7
D6WYN3	654942	serine-type protease inhibitor	8	35	3′	58,583	Q9VSC9	FBgn0035833	Satellite	625	1.7
D6WYA1	657913	Copia protein (Gag-int-pol protein)	8	36	3′	262	B6V6Z8	??	Satellite	196	0.5
D6WYC9	656718	Cmp-n-acetylneuraminic acid synthase	8	37	Inside		B5RJF3	FBgn0052220	Transposon	831	
D6WV42	656028	CG5080	8	38	Inside		Q7K3E2	FBgn0031313	Transposon	582	
D6WYA0	100142507	Beaten path	8	39	5′	7165	Q94534	FBgn0013433	Transposon	1181	
D6WUX6	662235	Putative uncharacterized protein	8	40	Inside		Q7KUK9	FBgn0036454	Transposon	440	
D6X0E1	654938	defective proboscis extension response	7	41	Inside		Q9VFD9	FBgn0038282	Satellite	722	2.0
D6WPX8	662021	Ribosome-releasing factor 2, mitochondrial	7	42	5′	17,480	Q9VCX4	FBgn0051159	Transposon	905	
A2AX72	662058	Gustatory receptor	7	42	3′	1581	Q9VPT1	FBgn0041250	Transposon	905	
D6WTD1	661895	similar to chitinase 6	7	43	Inside		Q9W2M7	FBgn0034580	Satellite	1440	4.0
D6WPE6	100142073	voltage-gated potassium channel	7	44	Inside		P17970	FBgn0003383	Transposon	814	
D2A2C6	663849	Putative uncharacterized protein	4	45	5′	9489	Q9V3S3	FBgn0013300	Satellite	549	1.5
D2A2D1	663875	Putative uncharacterized protein	4	45	3′	10,920	Q9W191	FBgn0034994	Satellite	549	1.5
D2A2I0	657017	Putative uncharacterized protein	4	46	5′	5820	Q8SZ28	FBgn0033786	Satellite	558	1.6
D2A2I1	657098	Ribonucleoside-diphosphate reductase	4	46	3′	7000	P48591	FBgn0012051	Satellite	558	1.6
D1ZZG6	660983	Kinesin-like protein	4	47	Inside		Q9VLW2	FBgn0031955	Transposon	508	
D2A2P8	100142595	PiggyBac transposable element	4	48	Inside		Q9VHL1	FBgn0037633	Transposon	377	
D6WB65	655028	E74	2	49	5′	60,525	P20105	FBgn0000567	Satellite	770	2.1
D6WB73	654962	organic cation transporter	2	49	3′	2638	Q7K3M6	FBgn0034479	Satellite	770	2.1
D6WBG8	659129	pre-mRNA-splicing helicase BRR2	2	50	3′	4811	Q9VUV9	FBgn0036548	Satellite	728	
D6WB14	658844	monophenolic amine tyramine	2	51	5′	7955	P22270	FBgn0004514	Transposon	567	
D6WB15	658769	Cuticular protein 47Ef	2	51	3′	16,173	A1Z8H7	FBgn0033603	Transposon	567	
D6WB29	657778	Endoprotease FURIN	2	52	Inside		P30432	FBgn0004598	Transposon	1045	
A8DIV5	657942	Nicotinic acetylcholine receptor subunit alpha11	2	53	5′	13,645	P25162	FBgn0004118	Transposon	1021	
D6WB29	657778	Endoprotease FURIN	2	53	3′	4875	P30432	FBgn0004598	Transposon	1021	
D6X3I9	661787	Transcription initiation factor IIF	10	54	5′	10,025	Q05913	FBgn0010282	Satellite	870	2.4
D6X3J1	661827	Putative uncharacterized protein	10	54	3′	6607			Satellite	870	2.4
D6X4P3	655389	Neutral alpha-glucosidase ab	10	55	Inside		Q7KMM4	FBgn0027588	Satellite	694	1.9
D6X3H5	661246	Neurexin-4	10	56	5′	2234	Q94887	FBgn0013997	Satellite	224	0.6
D6X3H7	661308	Succinate semialdehyde dehydrogenase	10	56	3′	14,901	Q9VBP6	FBgn0039349	Satellite	224	0.6
D6X4V6	655916	Tubby, putative	10	57	Inside		Q9VB18	FBgn0039530	Transposon	763	
D6X3J6	662034	Putative uncharacterized protein	10	58	5′	1015	Q9VEJ9	FBgn0038511	Satellite	564	1.6
D6X3J7	657069	cdc73 domain protein	10	58	3′	27,239	Q9VHI1	FBgn0037657	Satellite	564	1.6
D2A693	663231	lysine-specific demethylase 4B	6	59	Inside		Q9V6L0	FBgn0053182	Satellite	498	1.4
D2A490	659655	Facilitated trehalose transporter Tret1-2 homolog	6	60	Inside		Q8MKK4	FBgn0033644	Transposon	689	
D2A6I4	659728	Putative uncharacterized protein	6	61	5′	116,030	Q9W4G2	FBgn0260971	Transposon	764	
D2A6I6	659791	Putative uncharacterized protein	6	61	3′	4860	Q9VNB4	FBgn0037323	Transposon	764	
D2A3V0	657272	Fasciclin-3	6	62	5′	37,286	P15278	FBgn0000636	Satellite	281	0.8
D2A3V3	657421	LIM domain kinase 1	6	62	3′	21,789	Q8IR79	FBgn0041203	Satellite	281	0.8
D6W8F4	660322	Disco-related	x	63	Inside		Q9VXJ5	FBgn0042650	Satellite	530	1.5
D6W8D3	659123	PlexA	x	64	5′	1973	O96681	FBgn0025741	Transposon	848	
D6WGD2	659272	Aldose-1-epimerase	x	64	3′	6472	Q9VRU1	FBgn0035679	Transposon	848	
B3MMG1	657652	Neural-cadherin	5	65	Inside		O15943	FBgn0015609	Satellite	273	0.8
D6WNN6	658579	Transient receptor potential-gamma protein	5	66	5′	2547	Q9VJJ7	FBgn0032593	Transposon	894	
A3RE80	658661	Cardioacceleratory peptide receptor	5	66	3′	27,105	Q868T3	FBgn0039396	Transposon	894	
A1JUG2	661207	Ultraspiracle	5	67	Inside		P20153	FBgn0003964	Satellite	379	1.1
D6WNB3	656063	Y box protein	5	68	5′	14,993	O46173	FBgn0022959	Satellite	455	1.3
D6WNB6	656095	Peptide chain release factor 1	5	68	3′	350,365	Q9VK20	FBgn0032486	Satellite	455	1.3

A list of genes with gene identity numbers, gene name, chromosomal location, position, and distance relative to the associated TCAST-like element, as well as a list of TCAST-like elements, their types (satellite or transposon-like), total length in bp, and copy number of satellite repeats within an array are shown.

There were only three cases in which two different TCAST-like elements were associated with the same gene: gene D6X2C4 contains TCAST-like sequences no. 6 and 13 within introns, gene D6X2U7 is flanked at 5′ and 3′ end by sequences no. 5 and 7, respectively, whereas gene D6WB29 is located at 3′ end of the sequence no. 53 and has sequence no. 52 within an intron. All other TCAST-like elements were positioned near or within different genes. Thus in total, there were 101 genes found in the vicinity of TCAST-like elements. Characteristics of the genes associated with TCAST-like elements, including gene identity number, gene name and chromosomal location, position relative to the associated TCAST-like element, and distances between TCAST-like elements and genes, are shown in Table 1 Distances between TCAST-like elements and genes range from 262 nt (gene positioned at 3′ site of the sequence no. 36), to a maximal distance of 404,270 nt (gene positioned at 5′ site of the sequence no. 5).

### Characteristics of TCAST-like elements

#### TCAST satellite-like elements:

Sequence analysis of the 68 TCAST-like elements identified within the vicinity of genes enabled their classification into two groups. The first group contains partial TCAST satellite monomers or tandemly arranged elements, either complete or partial dimers, trimers, or tetramers (Table 1). The minimal size of satellite repeat was 203 nt (0.6 of complete TCAST monomer; sequence no. 15), whereas the maximal size was 1440 nt (four complete TCAST monomers; sequence no. 43; Table 1). In many sequences, two subtypes of TCAST satellite monomers were mutually interspersed: Tcast1a and Tcast1b. Tcast1b corresponds to the TCAST satellite consensus that was used as a query sequence (Ugarković *et al.* 1996), and Tcast1a corresponds to the TCAST subfamily described in Feliciello *et al.* 2011. Tcast1a and Tcast1b have an average homology of 79% and are of similar sizes at 362 bp and 377 bp respectively, but are characterized by a divergent, subfamily specific region of approximately 100 bp (Feliciello *et al.* 2011). There were 34 TCAST satellite-like elements found within or in the region of 53 genes. Lengths of TCAST satellite-like elements (Table 1), their exact start and end sites within genomic sequence and composition (supporting information, Table S1) are provided.

**Figure 1:**
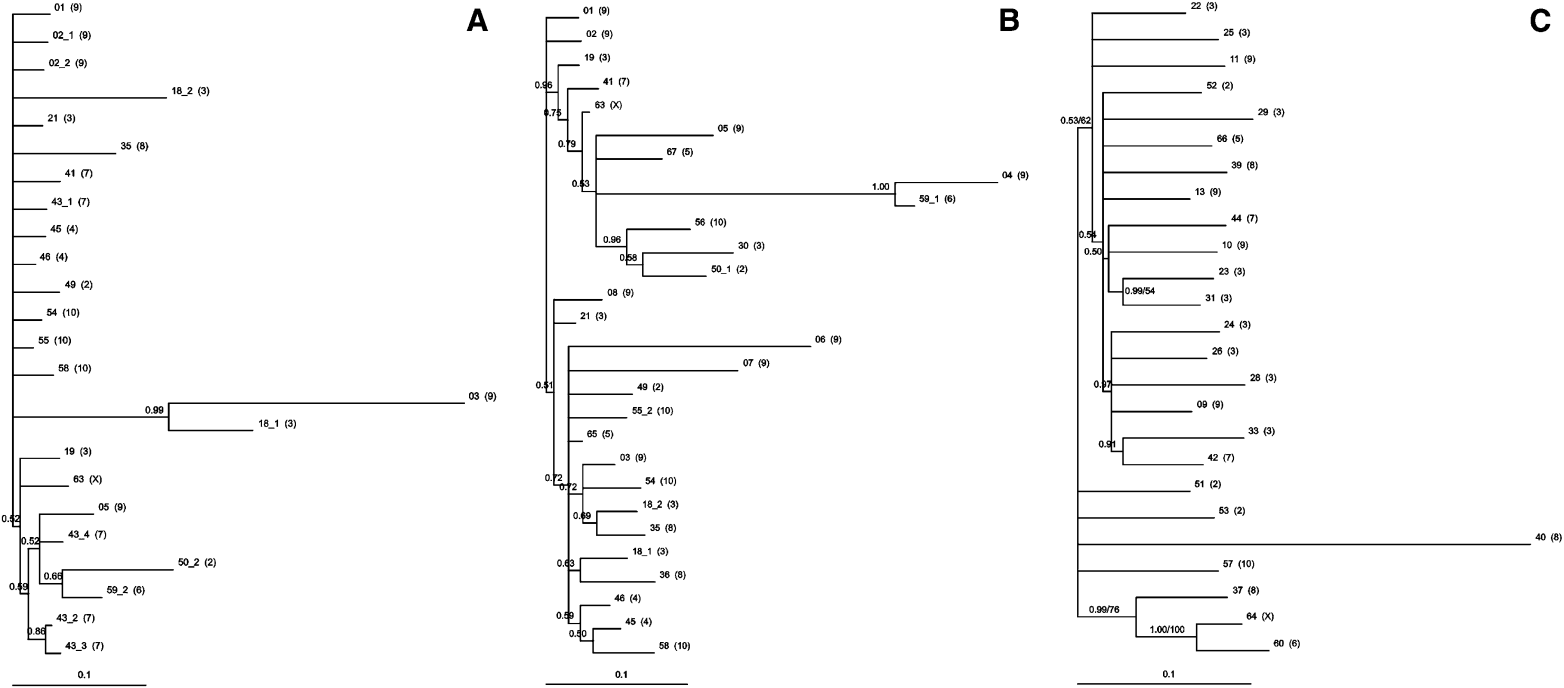
Bayesian/ML phylogenetic trees of: (A) TCAST satellite-like elements (subunits Tcast1a), (B) TCAST satellite-like elements (subunits Tcast1b), and (C) TCAST transposon-like elements. Sequence numbers correspond to those in Table 1. When a particular sequence is composed of few subrepeats (*e.g.*, Tcast1a or Tcast1b), numbers indicating subrepeats are added (*e.g.*, 43_1, 43_2, 43_3). Numbers in brackets indicate chromosomes on which the corresponding sequences are located. Numbers on branches indicate Bayesian posterior probabilities/ML bootstrap support (above 0.5/50%, respectively).

To see whether there is any clustering of sequences of TCAST satellite-like elements due to the difference in the homogenization at the level of local array, chromosome, or among different chromosomes, sequence alignment and phylogenetic analysis were performed. Tcast1a and Tcast1b subunits were extracted from TCAST satellite-like sequences and analyzed separately. Alignment was performed on 24 Tcast1a subunits, ranging in size from 136 and 377 bp (File S1). The average pairwise distances between Tcast1a subunits of TCAST satellite-like sequences was 5.8%. Alignment adjustment using Gblocks, which eliminates poorly aligned positions and divergent regions, resulted in few changes; therefore, the original, unadjusted alignment was used for the construction of phylogenetic trees. Because the sequences differ in lengths and comprise regions of divergent variability, methods that take into account specific models of DNA evolution were considered as the most suitable for the construction of phylogenetic trees, ML and Bayesian (Markov chain Monte Carlo). The ML tree showed weak resolution with no significant support for clustering of sequences derived from the same satellite-like array or from the same chromosome. Similarly, the Bayesian tree demonstrated no significant sequence clustering (Figure 1A).

Alignment of 28 Tcast1b subunits, ranging from 159 bp to 363 bp (File S2), was also not significantly affected by adjustment with Gblocks; therefore, the unadjusted alignment was used for the construction of phylogenetic trees (Figure 1B). The average pairwise divergence between Tcast1b subunits, of TCAST satellite-like sequences, was 6.7%. With the ML phylogenetic tree, four groups composed of two or three sequences, were resolved by relatively low bootstrap values. However, the majority of Tcast1b subunit sequences remained unresolved. There was no clustering of subunits derived from the same array or the same chromosome (Figure 1B). Bayesian tree analysis produced one significantly supported cluster composed of 10 sequences derived from 7 chromosomes (Figure 1B).

#### TCAST transposon-like elements:

**Figure 2:**
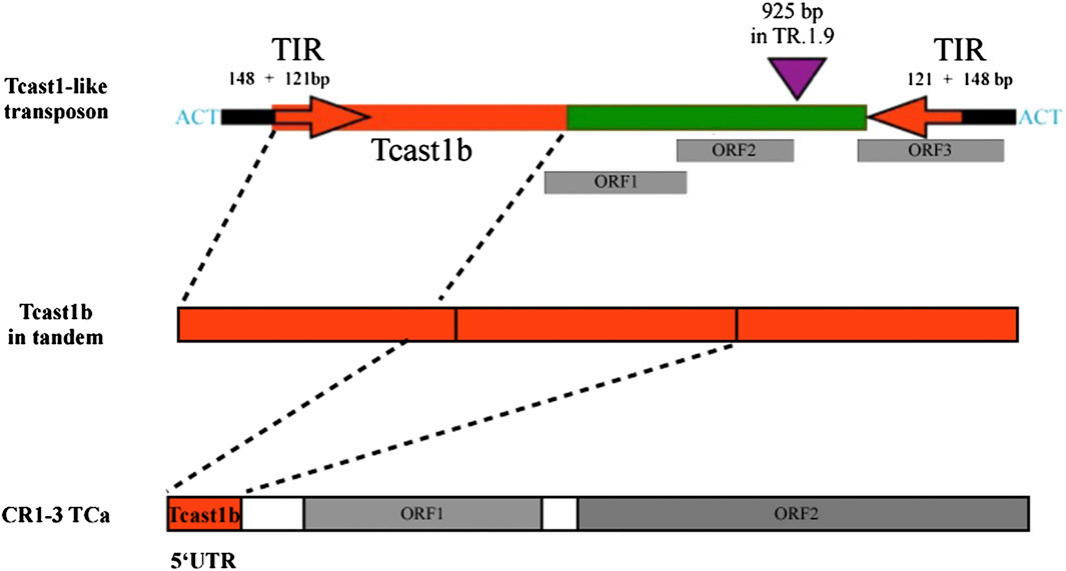
Organization of TCAST elements within *T. castaneum* genome in the form of TCAST transposon-like element, tandem arrays, and CR1-3_TCa retrotransposon. Regions corresponding to TCAST element are shown in red. TCAST transposon-like element contains an almost complete TCAST monomer and a monomer segment of approximately 121 bp in an inverted orientation, whereas CR1-3 retrotransposon contains segment corresponding to 1.2 monomer. Within TCAST transposon-like element terminal inverted repeats (arrows) unique nonsatellite sequence (green), target-site duplication in the form of “ACT,” and the insertion point of 925-bp sequence found within TR 1.9, element and coding for the putative transposase are shown. Three short ORFs within TCAST transposon-like element are also indicated. Within nonlong terminal repeat retrotransposon CR1-3_TCa regions corresponding to 5′UTR and to two ORFs are indicated.

The second group of TCAST-like repeats is represented by a complex element that contains an almost complete TCAST (or Tcast1b) monomer, and a TCAST monomer segment of approximately 121 bp in an inverted orientation. These two TCAST segments are separated by a nonsatellite sequence of approximately 306 bp. Both TCAST segments are part of TIRs that are approximately 269 bp long (Figure 2). As a result of the long TIRs, these elements are likely to form stable hairpin secondary structures and therefore resemble transposons. The nonsatellite part of sequence, common for all TCAST transposon-like elements, is unique in that it does not exhibit significant homology to any other sequence within the *T. castaneum* genome. There were 34 TCAST transposon-like elements found within or in the vicinity of 50 genes. Their lengths (Table 1) and exact start and end sites within genomic sequence (Table S1) are provided. Sequence analysis of TCAST transposon-like elements determined that 13 of them were > 1000 bp, with a maximal size of 1181 bp (Table 1). The remaining TCAST transposon-like elements were shorter, with a minimal size of 314 bp (sequence no. 27), and usually lacking part of, or one or both, TIRs. Conserved TIRs are necessary for transposition, and if they are absent, truncated, or mutated so that the transposase cannot interact with the transposon sequence, the transposon cannot be mobilized and therefore represents a molecular fossil of a once active transposon (Capy *et al.* 1998). Despite mutations and partial truncations of TIRs within the TCAST transposon-like elements, and likely because of the length of the TIRs, most of the elements still preserve a stable secondary structure and could potentially remain mobile.

Some TCAST transposon-like elements >1000 bp have a 3-bp duplication at the site of insertion in the form of ACT. One TCAST transposon-like element (sequence no. 39) is inserted into another repetitive DNA, indicated as Tcast2, which had been previously identified bioinformatically (Wang *et al.* 2008). Sequence analysis of this transposon-like element confirms the continuity of Tcast2 from the duplication site “ACT.” Typically, the size of target-site duplication is a hallmark of different superfamilies of eukaryotic DNA transposons, with *mariner/Tc1*, the only superfamily whose members are characterized by either 2- or 3-bp target-site duplication (Capy *et al.* 1998; Kapitonov and Jurka 2003; Feschotte and Pritham 2007). There are three open reading frames (ORFs) within TCAST transposon-like sequences, but the resulting putative proteins are very short and do not share similarity with any other proteins (Figure 2). The elements therefore do not code for transposases and are considered nonautonomous. Using the whole TCAST transposon-like elements as a query sequence, we searched the *T. castaneum* Gen Bank database for “full-sized” homologous elements that could potentially code for transposases and be considered autonomous. The search identified an element, named TR 1.9, with a 925-bp sequence inserted within a unique sequence of the TCAST transposon-like elements (Figure 2). This 925-bp sequence contains an ORF of 206 amino acids and a conserved domain belonging to the Transposase 1 superfamily, which also includes the mariner transposase. DNA transposons of the *mariner/Tc1* superfamily Mariner-1_TCa and Mariner-2_TCa, were identified within the *T. castaneum* genome (Jurka 2009a, 2009b). Using BLASTP and the translated sequence from the 925 bp ORF as a query sequence, we identified hits with a partial homology to a Mariner-2_TCa transposase and to a mariner-like element transposase present in two other insects, the beetle *Agrilus planipennis* (emerald ash borer**)** and *Chrysoperla plorabunda* (green lacewing; Neuroptera), but not to Mariner-1_ TCa transposase.

To test whether there is any chromosome-specific sequence clustering of TCAST transposon-like sequences that could suggest difference in homogenization within chromosome and among different chromosomes, the alignment and subsequent phylogenetic analysis of TCAST transposon-like sequences was performed. Because TCAST transposon-like elements differ significantly in size (314−1181 nt), the alignment and phylogenetic analyses was performed on 25 elements that mutually overlap in their sequences, whereas the other nine TCAST transposon-like elements were excluded from the analysis due to the very low overlapping with other elements. Alignment was additionally adjusted using Gblocks (File S3). The average pairwise divergence among TCAST transposon-like sequences was 12.7%. ML and Bayesian methods gave similar tree topologies (Figure 1C). The ML tree showed very weak resolution of TCAST transposon-like sequences and a general absence of subgroups with specific sequence characteristics (Figure 1C). Only two clusters were formed whereas, using the Bayesian tree, we identified three well-supported groups; two of them were as for ML tree (Figure 1C).

### Distribution of TCAST-like elements on *T. castaneum* chromosomes

**Figure 3:**
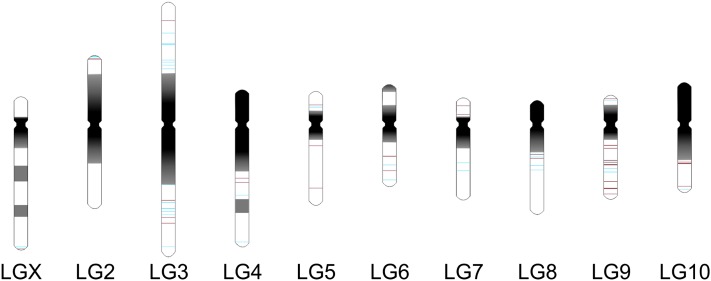
Distribution of TCAST-like elements on *T. castaneum* chromosomes. The karyotype representing the haploid set of *T. castaneum* chromosomes, and positions of constitutive heterochromatin (dark) and euchromatin (white) are depicted based on C-banding data (Stuart and Mocelin 1995) and *T. castaneum* 3.0 assembly (http://www.beetlebase.org). TCAST transposon-like elements (blue) and TCAST satellite-like elements (red) are shown. Two TCAST-like elements are represented as separate lines if they are at least 100 kb distant from each other.

TCAST-like elements found in the vicinity of genes were distributed on all 10 *T. castaneum* chromosomes (Table 1). Positions of constitutive heterochromatin and euchromatin were assigned on the haploid set of *T. castaneum* chromosomes, based on C-banding data (Stuart and Mocelin 1995) and *Tribolium castaneum* 3.0 Assembly data (Figure 3). Within euchromatic segments, the position of each TCAST-like element is specifically indicated (Figure 3) based on the position within the genomic sequence (Table S1). TCAST-like elements were dispersed on both arms of chromosomes 3, 5, 9, and 7, whereas on other chromosomes they were located on a single arm (Figure 3). The number of TCAST-like elements ranged from 2 on chromosome 1(X) to 17 on chromosomes 3 and 9. To detect whether TCAST-like elements were distributed randomly among the *T. castaneum* chromosomes or whether there was a significant over or underrepresentation of the elements on some chromosomes we performed hypergeometric distribution analysis test. The analysis revealed no statistically significant deviation in the number of TCAST-like elements among the chromosomes (Figure S1), pointing to their random distribution.

To determine whether there was a target preference for the insertion of TCAST-like elements, for example high AT content or another sequence characteristic, we analyzed the AT content within 100 bp of the flanking regions for each TCAST -like element, from both 5′ and 3′ sites (Figure S2 and Figure S3). The average AT content of the flanking regions for both TCAST satellite-like elements and TCAST transposon-like elements did not differ significantly from the average AT content of the whole *T. castaneum* genome or from the AT content of randomly selected intergenic regions and introns. Thus, this finding suggests that with regard to AT content, there is no target preference for the insertion of TCAST-like elements. Furthermore, alignment and comparison of all flanking sequences of TCAST-like elements did not identify any common sequence motifs.

### Genes in the vicinity of TCAST-like elements

Uniprot gene numbers were used as identifiers of genes located in the vicinity of TCAST-like elements (gene names shown in Table 1). Uniprot gene numbers for homologous genes found in *Drosophila melanogaster* are also indicated (Table 1). Detailed description of the genes, including molecular function of their protein products, biological processes in which these proteins are involved, and their cellular localization (cellular component), are shown (Table S1). Each identified gene is assigned to a particular TCAST-like element within its vicinity, and the precise position of TCAST-like elements in genomic sequence (start and end site) is indicated (Table S1). Functional analysis revealed that 17 of 101 genes correspond to putative uncharacterized proteins, whereas the remaining genes are involved in different molecular functions and diverse biological processes. Among the proteins, a proportion is characterized by ATP binding activity (13 proteins) and involvement in protein phosphorylation and /or signal transduction (9 proteins; Table S1).

To determine whether TCAST-like elements are distributed randomly relative to genes or whether they are overrepresented near specific groups of genes, we used GeneCodis 2.0 to provide a statistical representation of the genes associated with TCAST-like elements. Because many genes are still not annotated in *T. castaneum* and furthermore *T. castaneum* genomic data are not included in GeneCodis, we used gene numbers for orthologous genes from *D. melanogaster* for the analysis and compared them with the whole set of 14,869 genes annotated in *D. melanogaster*. Genecodis analysis revealed that TCAST-like elements are located near nine genes characterized as members of the immunoglobulin protein superfamily. Because there are only 134 immunoglobulin-like genes present within the total set of *D. melanogaster* genes, random distribution of TCAST-like elements would result in their occurrence near approximately a single immunoglobulin-like gene. The presence of TCAST-like elements in the vicinity of nine immunoglobulin-like genes therefore represents a statistically significant overrepresentation (0.00000427). All nine genes exhibit structural features of immunoglobulin-like, immunoglobulin subtype 1 and immunoglobulin subtype 2 proteins and are associated with the following TCAST transposon-like elements: 25 at the 3′end, 28 and 39 at the 5′ end, 32 and 40 within introns, and TCAST satellite-like elements: 8 at the 3′ end, 19 and 62 at the 5′ end, and 41 within intron (Table 1). A minimal distance between TCAST-like element and immunoglobulin-like gene was 7165 bp and a maximal 173,881 bp (Table 1). Molecular function of most of immunoglobulin-like genes is unknown, and they are involved in different biological processes such as cell adhesion, protein phosphorylation, and axon guidance (Table S1). Although all nine genes belong to immunoglobulin superfamily, they did not exhibit sequence similarity, which could suggest role of duplication in their evolution and spreading. The position of TCAST-like elements relative to the genes also was not consistent with the possibility that TCAST-like elements duplicated along with the immunoglobulin- like genes.

Overrepresentation of TCAST-like elements was also found near genes that exhibit ATP-binding activity and axon guidance properties but with a marginal significance (0.0183374 and 0.00865139). For the rest of genes, no significant overrepresentation of TCAST-like elements was detected. Thus, enrichment of TCAST-like elements in the vicinity of immunoglobulin-like genes potentially implicates a role of TCAST-like elements in the regulation of these genes.

## Discussion

TEs are classified in several dozen families based on transposition mechanisms and different dynamics properties (Hua-Van *et al.* 2005). Active TEs encode the enzymes necessary for their transposition, either to move between nonhomologous regions in the genome or to copy themselves to other positions. In many cases, TEs do not produce their own enzymes but are able to use those from functional copies or even from other TEs families. Defective and inactive TEs often are amplified in regions of low recombination such as heterochromatin and may form tandemly repeated satellite DNAs. The origin of satellite DNA array from transposon-like elements is reported for many insects such as *Drosophila melanogaster* (Agudo *et al.* 1999), *Drosophila guanche* (Miller *et al.* 2000), and the beetle *Misolampus goudoti* (Pons 2004) whereas the retroviral-like features were first observed in the satellite DNA from rodents of the genus *Ctenomys* (Rossi *et al.* 1993).

**Figure 4:**
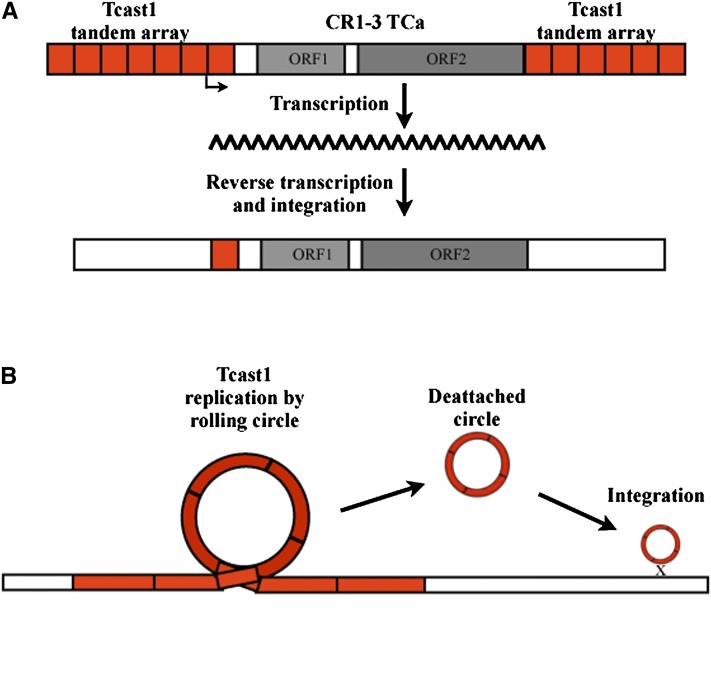
Models of spreading of TCAT-like elements based on (A) retrotransposition of CR-3_TCa element. CR1-3_TCa was inserted within TCAST satellite array and through recombination has acquired a part of TCAST sequence, which could act as a promoter and become a new functional 5′UTR. Subsequent retrotransposition of CR1-3_TCa could explain the dispersion of TCAST within the euchromatin. (B) Rolling circle replication of TCAST satellite DNA sequences excised from their heterochromatin loci via intrastrand recombination, followed by reintegration into different genome locations by homologous recombination.

Transposons can be inserted into other repetitive sequences such as satellite DNAs, as has been observed for the *mariner*-like element and MITE element, both inserted into satellite DNA of the ant *Messor bouvieri* (Palomeque *et al.* 2006). Searching for repetitive elements homologous to the TCAST repeat within Repbase (http://www.girinst.org/repbase/) revealed that 5′ UTR of nonlong terminal repeat retrotransposon CR1-3_TCa (Jurka 2009c) shares a high similarity of 83% with a 444-bp long TCAST sequence composed of 1.2 tandem monomers (Figure 1). Other CR1 subfamilies identified within *T. castaneum* such as CR1-1_ TCa, CR1-2_TCa, and CR1-4_TCa, published in Repbase, do not share similarity to CR1-3 and do not contain TCAST similar sequence. We propose that CR1-3 was inserted within TCAST satellite array and through recombination has acquired a part of TCAST sequence. Newly acquired TCAST element could act as a promoter because TCAST satellite DNA has an internal promoter for RNA Pol II (Pezer and Ugarković 2012) and becomes a new functional 5′ UTR. Subsequent retrotransposition of CR1-3_TCa could explain the dispersion of TCAST within the euchromatin (Figure 4). Three CR1-3_TCa elements with TCAST in the 5′UTR were identified within scaffolds that have not been mapped to linkage groups. However, truncated fragments with partial homology to CR1-3_TCa retrotransposon can be mapped within *T. castaneum* genome, some of them in the vicinity of TCAST elements. Such arrangement also indicates the role of CR1-3_TCa in the spreading of TCAST elements. There is also a possibility that TCAST satellite DNA originates from CR1-3 retrotransposon which was, after inactivation, amplified within the heterochromatin region. In the case of TCAST transposon-like elements, part of the satellite sequence is incorporated within TIRs which are characteristic for DNA transposons. The presence of target-site duplications at the sites of insertions of some TCAST transposon-like elements also indicates transposition as a mode of spreading of TCAST elements. Parts of satellite DNA elements can be found within some transposons, such as *pDv* transposon (Evgen'ev *et al.* 1982; Zelentsova *et al.* 1986) whose long direct terminal repeats show significant sequence similarity to the pvB370 satellite DNA, located in the centromeric heterochromatin of a number of species of the *Drosophila virilis* group (Heikkinen *et al.* 1995). The presence of short stretches of PisTR-A satellite DNA sequences within 3′ UTR of Ogre retrotransposons dispersed in the pea (*Pisum sativum*) genome was reported (Macas *et al.* 2009). Furthermore, the mobilization of subtelomeric repeats upon excision of the transposable *P* element from tandemly repeated subtelomeric sequences has been observed (Thompson-Stewart *et al.* 1994).

Incorporation of part of a TCAST satellite DNA sequence into a (retro)transposable element, and its subsequent mobilization and spreading by (retro)transposition, may explain the distribution of TCAST element in the vicinity of genes within euchromatin. Satellite DNA sequences are prone to undergo recurrent repeat copy number expansion and contraction in divergent lineages as well as among populations of the same species (Bosco *et al.* 2007). This amplification appears to be random and does not correlate with phylogeny of the species (Pons *et al.* 2004; Lee *et al.* 2005; Bulazel *et al.* 2007). Amplification of a satellite sequence is reported to occur as a result of unequal crossing over or duplicative transposition (Smith 1976; Ma and Jackson 2006). The discovery of human extrachromosomal elements originating from satellite DNA arrays in cultured human cells and different plant species indicates the possible existence of additional amplification mechanisms based on rolling-circle replication (Assum *et al.* 1993; Navrátilová *et al.* 2008). It has been proposed that satellite sequences excised from their chromosomal loci via intrastrand recombination could be amplified in this way, followed by reintegration of tandem arrays into the genome (Feliciello *et al.* 2006). Moreover, it is possible that such a mechanism affected TCAST satellite DNA, and that extrachromosamal circles of TCAST were reintegrated into different genome locations by homologous recombination based on short stretches of sequence similarity between TCAST satellite and target genomic sequence (Figure 4). Integrated TCAST sequences are mainly composed of interspersed elements belonging to two major subfamilies, Tcast1a and Tcast1b, which is a prevalent type of organization in pericentromeric heterochromatin (Feliciello *et al.* 2011). This finding indicates that the origin of dispersed euchromatic TCAST elements may be duplication of heterochromatin copies.

The distribution of TCAST-like elements relative to protein coding genes revealed no specific preference for insertions within introns or at 5′ or 3′ ends of genes. TCAST-like elements are distributed on all chromosomes with no significant deviation in the number among the chromosomes, and phylogenetic analysis did not detect any significant sequence clustering of TCAST-like elements derived from the same chromosome. Dispersed TCAST satellite-like elements produce tandem arrays up to tetramers, but repeats from the same array do not reveal any significant clustering on phylogenetic trees. This finding indicates there is no significant difference in the homogenization of TCAST satellite-like repeats at the level of local arrays or chromosome or among different chromosomes. The average pair-wise sequence divergence (6% for dispersed TCAST satellite-like repeats) is greater than the usual divergence of satellite elements located in heterochromatin of tenebrionid beetles [approximately 2% (Ugarković *et al.* 1996)]. This difference in homogeneity between repeats located in heterochromatin and euchromatin may be explained by a lower rate of gene conversion affecting dispersed satellite-like elements or by a specific mechanism of DNA repair acting on satellite DNA (Feliciello *et al.* 2006). TCAST transposon-like elements dispersed among the genes within euchromatin have an even greater average sequence divergence (approximately 12%) and also exhibit no significant chromosome-specific sequence clustering, indicating a similar rate of homogenization within and among the chromosomes. Relatively high sequence divergence of TCAST transposon-like elements and the significant truncation of the majority of them, indicates that the transposition of these elements did not occur very recently and that these elements could be considered as molecular fossils of the functional TCAST transposon-like elements.

*Cis*-regulatory elements, such as promoters or transcription factor binding sites, are predicted in some satellite DNAs (Pezer *et al.* 2011). Transcription from promoters for RNA Pol II is also characteristic for pericentromeric satellite DNAs from the beetles *Palorus ratzeburgii* and *Palorus subdepressus* (Pezer and Ugarković 2008, 2009). Temperature-sensitive transcription of TCAST satellite DNA from an internal RNA Pol II promoter has been demonstrated (Pezer and Ugarković 2012). Based on these findings, it can be proposed that TCAST elements located in the vicinity of genes may function as alternative promoters, and transcripts derived from them may interfere with the expression of neighboring gene. This type of regulation is often observed for retrotransposons positioned immediately 5′ of protein genes (Faulkner *et al.* 2009). In addition, some tissue-specific gene promoters are derived from retrotransposons (Ting *et al.* 1992; Samuelson *et al.* 1996). Because of rapid evolutionary turnover, satellite DNA sequences often are restricted to a group of closely related species, or in some instances are species specific. This is the case with TCAST satellite DNA, which is not even detected in the congeneric *Tribolium* species. If restricted satellite DNAs have regulatory potential, then insertion of these elements in vicinity of genes could contribute to the establishment of lineage-specific or species-specific patterns of gene expression. Annotation of genes in proximity to TCAST-like elements demonstrated a statistical overrepresentation of certain groups of genes, for example, those with immunoglobulin-like domains. Recently, in the fish *Salvelinus fontinalis*, a regulatory role of a 32-bp satellite repeat, located in an intron of the major histocompatibility complex gene (MHIIβ), on MHIIβ gene expression was demonstrated (Croisetiere *et al.* 2010). The level of gene expression depends on temperature, as well as the number of satellite repeats, and indicates a role for temperature-sensitive satellite DNA in gene regulation of the adaptive immune response. Further studies are necessary to determine whether TCAST-like elements exhibit a potential regulatory role on nearby genes. The transcriptional potential of satellite DNAs as well as their distribution close to protein-coding genes, as shown in this study, provides strong support, that in addition to transposons, satellite DNAs represent a rich source for the assembly of gene regulatory systems.

## Supplementary Material

Supporting Information
