# Supplementary material for: Satellite DNA-Like Elements Associated With Genes Within Euchromatin of the Beetle Tribolium castaneum
Source: G3 (Bethesda). 2012 Aug 1;2(8):931–41. doi: 10.1534/g3.112.003467 (PMC3411249; doi:10.1534/g3.112.003467)
Supplement: Supporting Information [file supp_2_8_931__index.html]

Supporting Information 

# Satellite DNA-Like Elements Associated With Genes Within Euchromatin of the Beetle *Tribolium castaneum*

## Supporting Information for Brajkovic *et al.*, 2012

**Files in this Data Supplement:**

- Supporting Information - Figures S1-S3, Table S1, and Files S1-S3 (PDF, 461 KB)
- Figure S1 - In each chromosome the frequency of TCAST-like elements is compared with the frequency in the complete sample and deviations are shown by log-odds (y-axis) (PDF, 183 KB)
- Figure S2 - AT content within 100 bp of the flanking regions for each of TCAST satellite-like elements, both from 5' (blue) and 3' site (green), and from each TCAST satellite-like element (red) (PDF, 138 KB)
- Figure S3 - AT content within 100 bp of the flanking regions for each of TCAST satellite-like elements, both from 5' (blue) and 3' site (green), and from each TCAST transposone-like element (red) (PDF, 128 KB)
- Table S1 - Chromosomal location, exact start and end site, and composition of TCAST-like elements within genomic sequence (PDF, 174 KB)
- File S1 - Alignment of TCAST satellite-like elements subunits Tcast1a (.ppt, 1.1 MB)
- File S2 - Alignment of TCAST satellite-like elements subunits Tcast1b (.ppt, 1.3 MB)
- File S3 - Alignment of TCAST transposon-like elements (.ppt, 2.2 MB)
