# Supplementary material for: Satellite DNA-Like Elements Associated With Genes Within Euchromatin of the Beetle Tribolium castaneum
Source: G3 (Bethesda). 2012 Aug 1;2(8):931–41. doi: 10.1534/g3.112.003467 (PMC3411249; doi:10.1534/g3.112.003467)
Supplement: Supporting Information [file supp_2.8.931_003467SI.pdf]

TCAST-like elements

Chromosome	1	2	3	4	5	6	7	8	9	10	
RawP_value	ns	ns	ns	ns	ns	ns	ns	ns	ns	ns	total
Sat_elements	2	5	17	4	4	4	4	6	17	5	68

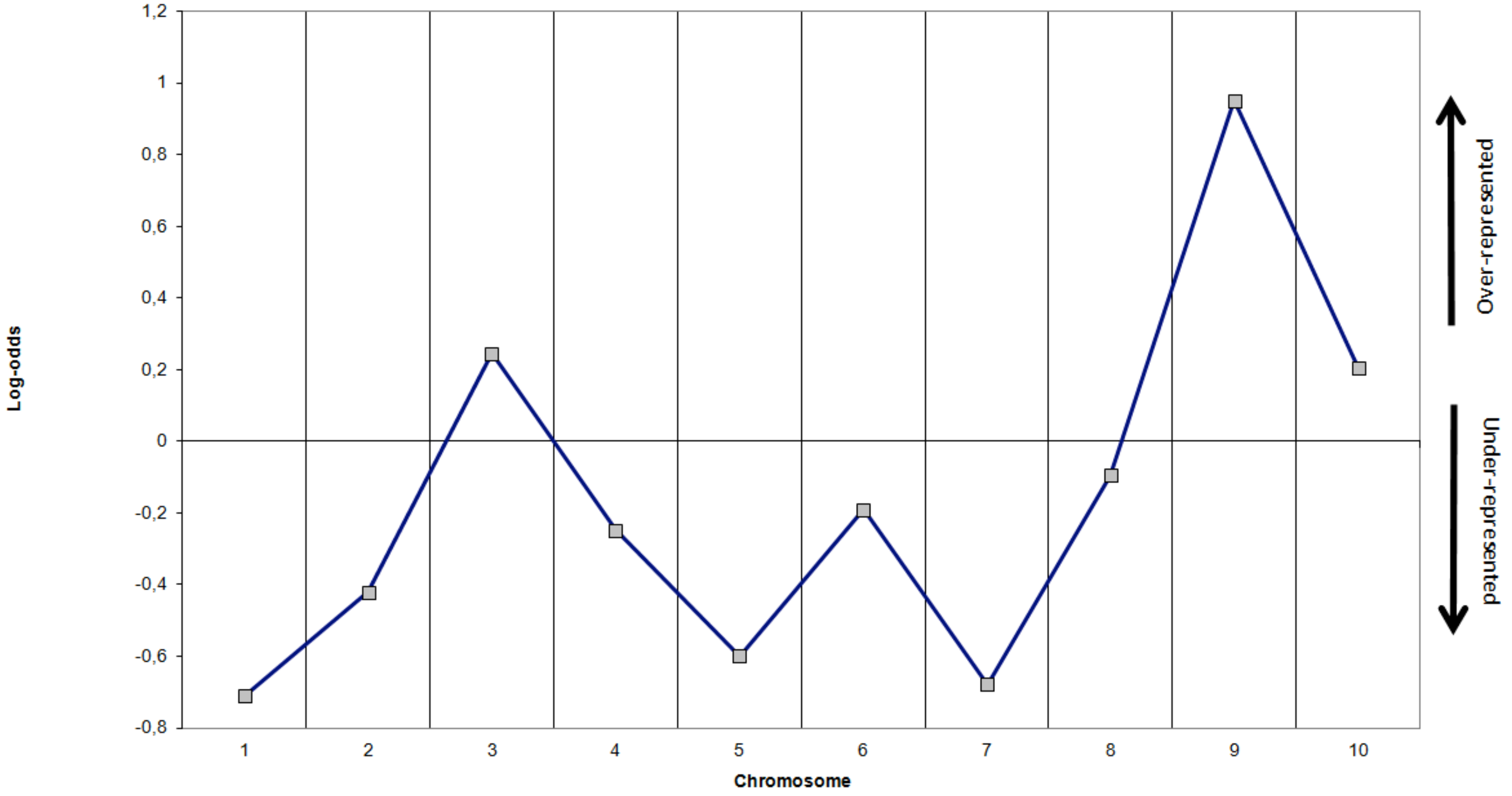

Figure S1 In each chromosome the frequency of TCAST-like elements is compared with the frequency in the complete sample and deviations are shown by log-odds (y-axis). Log-odds of zero denotes that the frequency of TCAST-like elements in chromosome and in the complete sample do not differ, whereas positive and negative values point to over-representation and under-representation, respectively. Significance of the deviations is shown in the p-value chart..

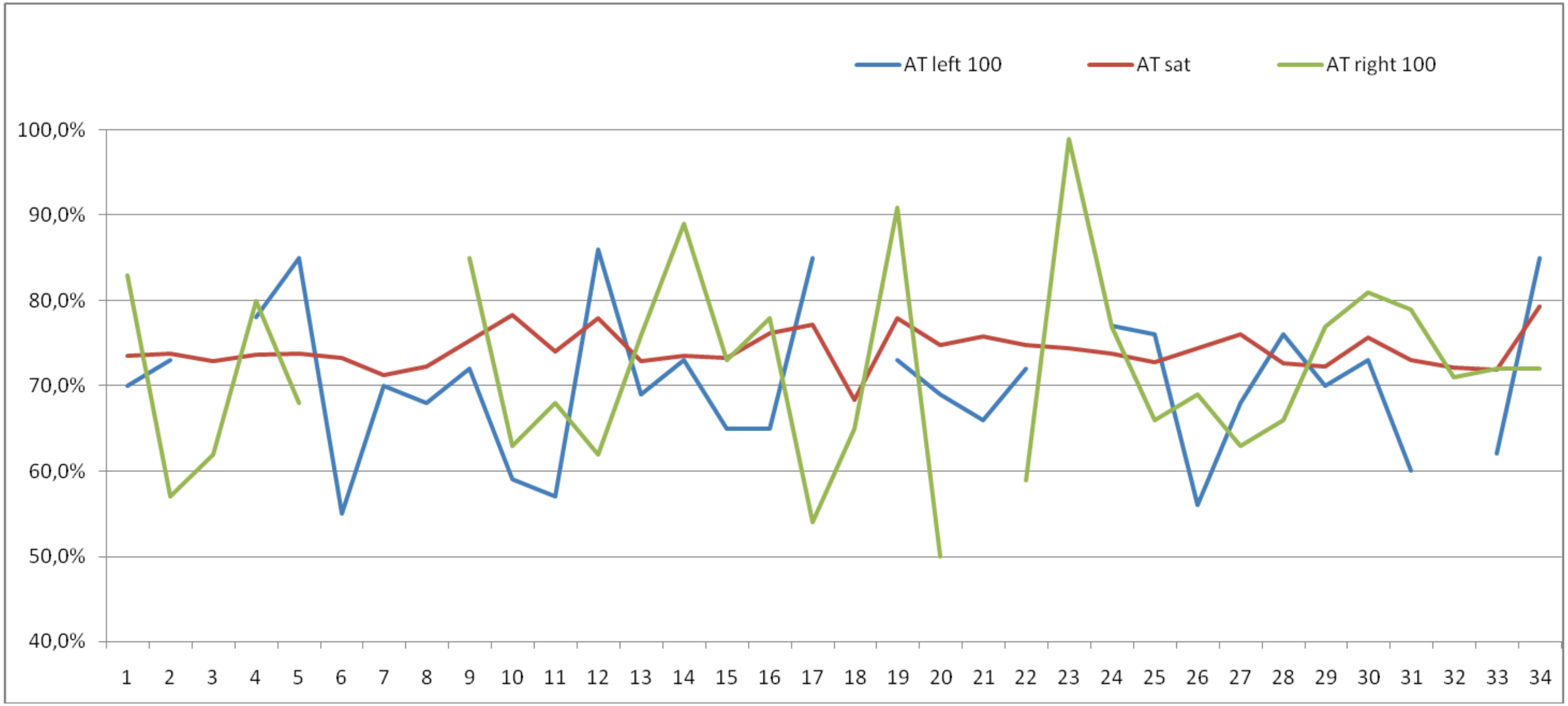

Figure S2 AT content within 100 bp of the flanking regions for each of TCAST satellite-like elements, both from 5’ (blue) and 3’ site (green), and from each TCAST satellite-like element (red).

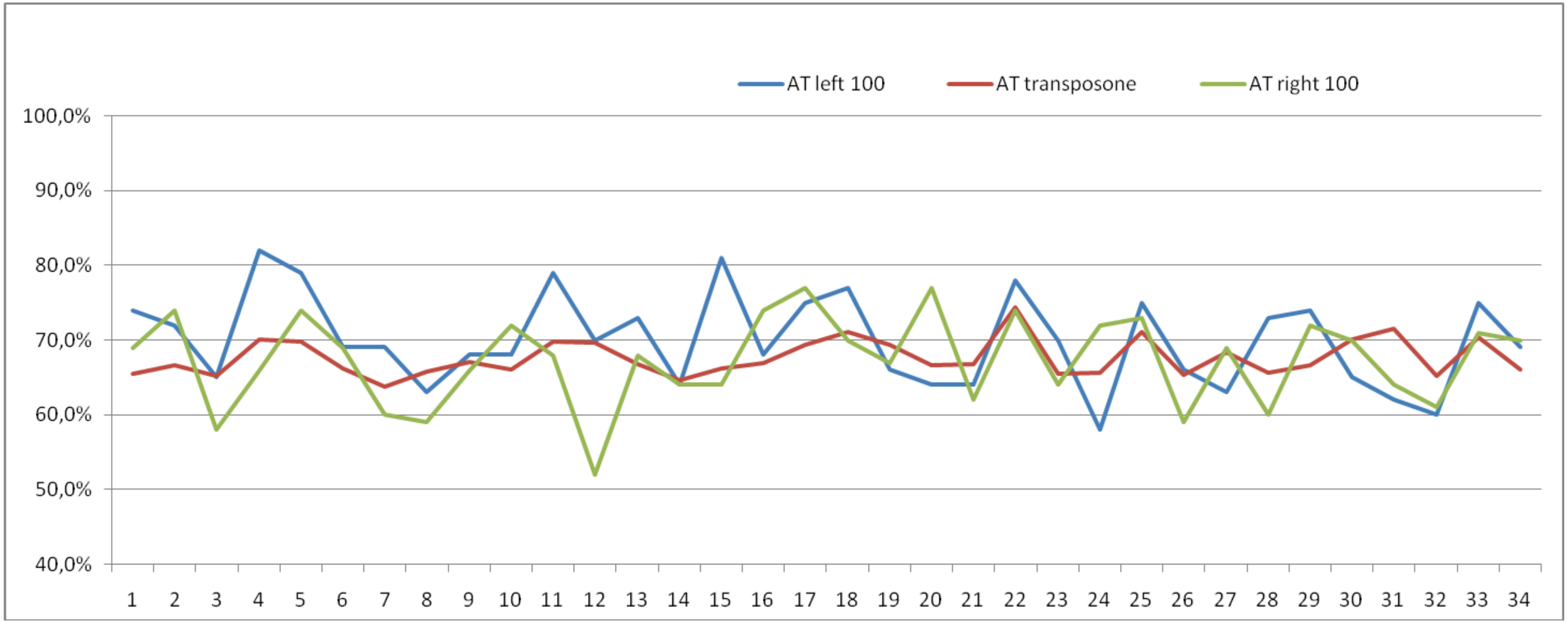

Figure S3 AT content within 100 bp of the flanking regions for each of TCAST satellite-like elements, both from 5' (blue) and 3' site (green), and from each TCAST transposone-like element (red).

File S1

Alignment of TCAST satellite-like elements subunits Tcast1a

File S1 is available for download as a ppt file at http://www.g3journal.org/lookup/suppl/doi:10.1534/g3.112.003467/-/DC1

File S2

Alignment of TCAST satellite-like elements subunits Tcast1b

File S2 is available for download as a ppt file at http://www.g3journal.org/lookup/suppl/doi:10.1534/g3.112.003467/-/DC1

File S3

Alignment of TCAST transposon-like elements

File S3 is available for download as a ppt file at http://www.g3journal.org/lookup/suppl/doi:10.1534/g3.112.003467/-/DC1

For TCAST transposon-like elements (File S1), alignment was additionally adjusted using Gblocks which eliminates poorly aligned positions and divergent regions.

Table S1 Chromosomal location, exact start and end site, and composition of TCAST-like elements within genomic sequence

Name	Variant	Location	Start	End	Genome View
1	BA	LG9	18138275	18139008	**(left)** Distant approximately 19kb from gene "D6WZP1" .<br>**Molecular function:** ATP binding; protein kinase activity.<br>**Biological process:** protein phosphorylation.<br>**Celular component:** unknown.<br><br>**(right)** Distant approximately 8kb from gene "D6WZP3 (Q9VP48)" .<br>**Molecular function:** GTP binding .<br>**Biological process:** protein transport; small GTPase mediated signal transduction.<br>**Celular component*:** intrinsic to plasma membrane.
2	ABA	LG9	17975173	17976213	**Transcriptin:** Part of transcript from gene "D6WZL9".<br>**Molecular function:** ATP binding; protein serine/threonine kinase activity; transferase activity, transferring phosphorus-containing groups.<br>**Biological process:** protein phosphorylation.<br>**Celular component:** unknown.
3	AB	LG9	9281520	9282235	**(left)** Distant approximately 100kb from gene "D6X226 (Q8IP89)" .<br>**Molecular function:** nucleotide binding; RNA binding.<br>**Biological process*:** regulation of alternative nuclear mRNA splicing, via spliceosome; inter-male aggressive behavior; mRNA polyadenylation; germ cell development; spermatid development; oogenesis; positive regulation of exit from mitosis; germ-line stem cell division; negative regulation of oskar mRNA translation. **Celular component*:** nucleus; P granule.<br><br>**(right)** Distant approximately 116kb from gene "D6X238 (P16554)" .<br>**Molecular function***: protein binding; Notch binding; nucleotide binding; ATP binding.<br>**Biological process*:** cell fate determination; neuroblast fate determination; central nervous system development; heart development; pericardial cell differentiation; protein localization; multicellular organismal development; glial cell migration; regulation of asymmetric cell division; sensory organ precursor cell division; negative regulation of Notch signaling pathway; muscle cell fate specification; regulation of neurogenesis, asymmetric neuroblast division.<br>**Celular component*:** nucleus; cytoplasm; cell cortex; basal part of cell; basal cortex.
4	AB	LG9	10699909	10700425	**(left)** Distant approximately 1,5kb from gene "hypotetical protein" .<br>**Molecular function**: unknown.<br>**Biological process:** unknown .<br>**Celular component:** unknown.<br><br>**(right)** Distant approximately 6,5kb from gene "D6X2D0".<br>**Molecular function:** oxidoreductase activity; nucleotide binding.

Name	Variant	Location	Start	End	Genome View
					**Biological process:**oxidation-reduction process .<br>**Celular component:** unknown.
5	AgBA	LG9	4568301	4569358	**(left)** Distant approximately 404kb from gene "D6X1E7 (Q9VWR5)" .<br>**Molecular function:** monooxygenase activity; iron ion binding; electron carrier activity; oxidoreductase activity, acting on paired donors, with incorporation or reduction of molecular oxygen; heme binding. **Biological process:** oxidation-reduction process.<br>**Celular component*:** microsome; endoplasmic reticulum membrane.<br><br>**(right)** Distant approximately 10kb from gene "D6X2U7".<br>**Molecular function:** unknown.<br>**Biological process:** unknown.<br>**Celular component:** integral to membrane.
6	B	LG9	10553279	10553582	**Transcriptin:** Part of transcript from gene "D6X2C4".<br>**Molecular function:** signal transducer activity; dopamine receptor activity; G-protein coupled receptor activity.<br>**Biological process:** signal transduction; G-protein coupled receptor protein signaling pathway; dopamine receptor signaling pathway.<br>**Celular component:** integral to membrane.
7	B	LG9	4587153	4587546	**(left)** Distant approximately 7kb from gene "D6X2U7".<br>**Molecular function:** unknown.<br>**Biological process:** unknown.<br>**Celular component:** integral to membrane.<br><br>**(right)** Distant approximately 50kb from gene "D6X366".<br>**Molecular function:** unknown.<br>**Biological process:** unknown.<br>**Celular component:** integral to membrane.
8	B	LG9	19746866	19747078	**(left)** Distant approximately 57kb from gene "D6X0D7".<br>**Molecular function:** nucleotide binding; ATP binding; protein tyrosine kinase activity; calcium ion binding.<br>**Biological process:** protein phosphorylation; homophilic cell adhesion.<br>**Celular component:** membrane.<br><br>**(right)** Distant approximately 63kb from gene "D6X0E1 (Q9VFD9)".<br>**Molecular function:** unknown.<br>**Biological process*:** behavioral response to ethanol.<br>**Celular component:** unknown.

Name	Variant	Location	Start	End	Genome View
9	TR	LG9	11855262	11856368	**Transcriptin:** Part of transcript from gene "D6X2H8".<br>**Molecular function:** metalloendopeptidase activity; zinc ion binding.<br>**Biological process:** proteolysis.<br>**Celular component:** unknown.
10	TR	LG9	13938275	13939359	**(left)** Distant approximately 48kb from gene "D6X2U7".<br>**Molecular function:** unknown.<br>**Biological process:** unknown.<br>**Celular component:** integral to membrane.<br><br>**(right)** Distant approximately 68kb from gene "D6X2V3 (Q9VDB7)".<br>**Molecular function:** unknown.<br>**Biological process*:** phagocytosis, engulfment.<br>**Celular component:** unknown.
11	TR	LG9	9763147	9764208	**Transcriptin:** Part of transcript from gene "D6X244 (Q0KID3)".<br>**Molecular function:** ATP binding; protein serine/threonine kinase activity; transferase activity, transferring phosphorus-containing groups.<br>**Biological process*:** protein phosphorylation; actin filament organization; regulation of cell shape.<br>**Celular component:** unknown.
12	BA	LG9	15894092	15894383	**Transcriptin:** Part of transcript from gene "D6X374".<br>**Molecular function:** unknown.<br>**Biological process:** unknown.<br>**Celular component:** unknown.
13	TR	LG9	10548788	10549687	**Transcriptin:** Part of transcript from gene " D6X2C4 (P41596)".<br>**Molecular function:** signal transducer activity; receptor activity; G-protein coupled receptor activity; dopamine receptor activity.<br>**Biological process:** signal transduction; G-protein coupled receptor signaling pathway; dopamine receptor signaling pathway; thermotaxis*, associative learning*; visual learning*.<br>**Celular component:** integral to membrane.
14	AB	LG9	9894630	9894851	**(left)** Distant approximately 9,5kb from gene "D6X259"<br>**Molecular function:** transferase activity, transferring glycosyl groups.<br>**Biological process:** metabolic process.<br>**Celular component:** unknown.<br><br>**(right)** Distant approximately 34kb from gene "D6X260 (Q9VYB7)"

Name	Variant	Location	Start	End	Genome View
					**Molecular function:** sulfotransferase activity; protein-tyrosine sulfotransferase activity*.<br>**Biological process*:** protein secretion.<br>**Celular component*:** integral to membrane; Golgi membrane.
15	AB	LG9	1062251	1062452	**(left)** Distant approximately 40kb from gene "D6X075 (Q9VU34)"<br>**Molecular function:** zinc ion binding.<br>**Biological process:** unknown.<br>**Celular component:** unknown.<br><br>**(right)** Distant approximately 143kb from gene "D6X1P2 (Q9U3V5)"<br>**Molecular function:** zinc ion binding; DNA binding*.<br>**Biological process* :** regulation of transcription, DNA-dependent; multicellular organismal development; specification of segmental identity, head; epidermis morphogenesis; compound eye development.<br>**Celular component:** intracellular; nucleus*.
16	TR	LG9	1560153	1560740	**(left)** Distant approximately 4kb from gene "D6X095 (P47947)"<br>**Molecular function:** calcium ion binding.<br>**Biological process:** unknown.<br>**Celular component:** unknown.<br><br>**(right)** Distant approximately 15kb from gene "D6X0I1 (P47947)"<br>**Molecular function:** calcium ion binding.<br>**Biological process:** unknown.<br>**Celular component:** unknown.
17	AB	LG9	5501464	5501685	**Transcriptin:** Part of transcript from gene "D6X1J0 (Q9NB97)".<br>**Molecular function:** neurotransmitter:sodium symporter activity; dopamine transmembrane transporter activity*; cocaine binding*.<br>**Biological process:** neurotransmitter transport; dopamine transport*; sleep*; circadian sleep/wake cycle*.<br>**Celular component:** integral to membrane.
18	ABBA	LG3	23706992	23708337	**(left)** Distant approximately 126kb from gene "D6WF56 (Q7KAH0)"<br>**Molecular function:** nucleic acid binding; zinc ion binding.<br>**Biological process*:** regulation of chromatin silencing.<br>**Celular component:** intracellular.<br><br>**(right)** Distant approximately 64kb from gene "D6WF61 (Q9VHY5)"<br>**Molecular function*:** sequence-specific DNA binding transcription factor activity.<br>**Biological process:** transcription initiation from RNA polymerase II promoter.

Name	Variant	Location	Start	End	Genome View
					**Celular component:** transcription factor TFIID complex; nucleus.
19	BA	LG3	28288889	28289575	**(left)** Distant approximately 115kb from gene "D6WGB1 (P20241)"<br>**Molecular function:** calcium ion binding.<br>**Biological process*:** neuron cell-cell adhesion; epidermal growth factor receptor signaling pathway; multicellular organismal development; axonogenesis; imaginal disc morphogenesis; axon ensheathment; mushroom body development; septate junction assembly; nerve maturation; melanotic encapsulation of foreign target; regulation of tube size, open tracheal system; axon extension; dendrite morphogenesis; synapse organization; establishment of glial blood-brain barrier.<br>**Celular component*:** plasma membrane; pleated septate junction; tight junction; lateral plasma membrane; filopodium.<br><br>**(right)** Distant approximately 82kb from gene "D6WGB5 (P54611)"<br>**Molecular function:** proton-transporting ATPase activity, rotational mechanism.<br>**Biological process:** ATP hydrolysis coupled proton transport.<br>**Celular component:** proton-transporting two-sector ATPase complex, catalytic domain.
20	TR	LG3	36078931	36079422	**(left)** Distant approximately 4kb from gene "D6WII0 (Q9W3N7)"<br>**Molecular function:** NADH dehydrogenase activity.<br>**Biological process*:** oxidation-reduction process.<br>**Celular component:** mitochondrion.<br><br>**(right)** Distant approximately 18,5kb from gene "D6WII2"<br>**Molecular function:** unknown.<br>**Biological process:** unknown.<br>**Celular component:** unknown.
21	BA	LG3	4957720	4957084	**Transcriptin:** Part of transcript from gene "D6WFT8 (Q960Y9)".<br>**Molecular function*:** DNA binding.<br>**Biological process*:** mitotic chromosome condensation; gene silencing; heterochromatin formation; chromosome organization.<br>**Celular component*:** chromosome, centromeric region; heterochromatin; condensed chromosome; polytene chromosome; nuclear heterochromatin.
22	TR	LG3	17823306	17824305	**(left)** Distant approximately 10,5kb from gene "D6WDY2 (Q8SXC2)"<br>**Molecular function:** catalytic activity; transferase activity, transferring nitrogenous groups; pyridoxal phosphate binding; kynurenine-oxoglutarate transaminase activity*; 1-aminocyclopropane-1-carboxylate synthase activity*.<br>**Biological process:** biosynthetic process; 1-aminocyclopropane-1-carboxylate biosynthetic process*.<br>**Celular component:** unknown.<br><br>**(right)** Distant approximately 11,5kb from gene "D6WDY4 (P22464)"

Name	Variant	Location	Start	End	Genome View
					**Molecular function:** calcium ion binding; calcium-dependent phospholipid binding.<br>**Biological process*:** wing disc dorsal/ventral pattern formation.<br>**Celular component:** unknown.
23	TR	LG3	25914814	25915894	**Transcriptin:** Part of transcript from gene "D6WFK8 (Q7KU95)".<br>**Molecular function:** unknown.<br>**Biological process:** signal transduction; microtubule cytoskeleton organization*; neuromuscular junction development*; axon extension.<br>**Celular component*:** neuromuscular junction; presynaptic membrane; terminal button.
24	TR	LG3	26709980	26710867	**(left)** Distant approximately 64kb from gene "D6WFX1"<br>**Molecular function:** guanyl-nucleotide exchange factor activity.<br>**Biological process:** small GTPase mediated signal transduction.<br>**Celular component:** intracellular.<br><br>**(right)** Distant approximately 4kb from gene "D6WFX3"<br>**Molecular function:** catalytic activity; UDP-glucose:hexose-1-phosphate uridylyltransferase activity; zinc ion binding.<br>**Biological process:** galactose metabolic process.<br>**Celular component:** unknown.
25	TR	LG3	15992929	15993944	**(left)** Distant approximately 15kb from gene "D6WDQ4"<br>**Molecular function:** unknown.<br>**Biological process:** unknown.<br>**Celular component:** unknown.<br><br>**(right)** Distant approximately 9kb from gene "D6WDQ6 (A1ZA72)"<br>**Molecular function*:** protein serine/threonine kinase activity; calmodulin-dependent protein kinase activity; myosin light chain kinase activity; transferase activity.<br>**Biological process*:** protein phosphorylation.<br>**Celular component*:** microtubule associated complex.
26	TR	LG3	24245475	24246541	**(left)** Distant approximately 26kb from gene "D6WF68"<br>**Molecular function:** choline dehydrogenase activity; oxidoreductase activity, acting on CH-OH group of donors; flavin adenine dinucleotide binding.<br>**Biological process:** alcohol metabolic process; oxidation-reduction process.<br>**Celular component:** unknown.<br><br>**(right)** Distant approximately 93kb from gene "C3XZ92"<br>**Molecular function:** nucleotide binding; ATP binding; protein kinase activity; protein serine/threonine kinase activity; small

Name	Variant	Location	Start	End	Genome View
					GTPase regulator activity; transferase activity, transferring phosphorus-containing groups.<br>**Biological process:** protein phosphorylation.<br>**Celular component:** unknown.
27	TR	LG3	19520989	19521302	**Transcriptin:** Part of transcript from gene " D6WE82 (Q9VDK2)".<br>**Molecular function*:** nucleotide binding.<br>**Biological process:** unknown.<br>**Celular component:** unknown.
28	TR	LG3	11005355	11006180	**(left)** Distant approximately 174kb from gene "D6WHX6"<br>**Molecular function:** unknown.<br>**Biological process:** unknown.<br>**Celular component:** unknown.<br><br>**(right)** Distant approximately 83kb from gene "D6WI58 (Q95029)"<br>**Molecular function:** peptidase activity; cysteine-type endopeptidase activity; hydrolase activity.<br>**Biological process:** proteolysis; multicellular organismal development*; digestion*; autophagic cell death*; salivary gland cell autophagic cell death*.<br>**Celular component*:** lysosome; fusome.
29	TR	LG3	15448171	15449494	**(left)** Distant approximately 174kb from gene "D6WDJ9"<br>**Molecular function:** unknown.<br>**Biological process:** unknown.<br>**Celular component:** unknown.<br><br>**(right)** Distant approximately 384kb from gene "D6WDN0 (Q9U6Y9)"<br>**Molecular function:** methyltransferase activity; protein methyltransferase activity*; transferase activity*; protein-arginine omega-N symmetric methyltransferase activity*.<br>**Biological process:** methylation; transcription, DNA-dependent*; multicellular organismal development*; pole plasm assembly*; pole plasm protein localization*; intracellular mRNA localization*; peptidyl-arginine methylation*; peptidyl-arginine methylation, to symmetrical-dimethyl arginine*; cell differentiation*; P granule organization*; ecdysone receptor-mediated signaling pathway*; growth*; oogenesis*.<br>**Celular component:** cytoplasm; nucleus*.
30	BAB	LG3	29785393	29786204	**(left)** Distant approximately 38kb from gene "D6WGS3"<br>**Molecular function:** unknown.<br>**Biological process:** unknown.<br>**Celular component:** unknown.

Name	Variant	Location	Start	End	Genome View
					**(right)** Distant approximately 227kb from gene "D6WGT0 (Q11002)"<br>**Molecular function:** calcium ion binding; calcium-dependent cysteine-type endopeptidase activity; hydrolase activity*.<br>**Biological process:** proteolysis; phagocytosis, engulfment*; dorsal/ventral pattern formation*; protein autoprocessing*; BMP signaling pathway involved in spinal cord dorsal/ventral patterning*; cuticle development*.<br>**Celular component:** intracellular; cytoplasm*; actin cytoskeleton*; neuronal cell body*.
31	TR	LG3	16844007	16845066	**(left)** Distant approximately 379kb from gene "D6WDS8"<br>**Molecular function:** unknown.<br>**Biological process:** unknown.<br>**Celular component:** unknown.<br><br>**(right)** Distant approximately 8kb from gene "D6WDT0"<br>**Molecular function:** clathrin binding; phospholipid binding; 1-phosphatidylinositol binding.<br>**Biological process:** clathrin coat assembly.<br>**Celular component:** clathrin coat.
32	TR	LG3	8278354	8279019	**Transcriptin:** Part of transcript from gene "D6WHF2 (Q9W4T9)".<br>**Molecular function*:** protein binding.<br>**Biological process*:** compound eye morphogenesis; homophilic cell adhesion; heterophilic cell-cell adhesion; garland cell differentiation; myoblast fusion; larval visceral muscle development; regulation of striated muscle tissue development.<br>**Celular component:** membrane; plasma membrane*; adherens junction*; cell surface*.
33	TR	LG3	11368959	11370016	**Transcriptin:** Part of transcript from gene "D6WI96 (P11147)".<br>**Molecular function:** nucleotide binding; ATP binding; protein binding*; chaperone binding*.<br>**Biological process*:** nuclear mRNA splicing, via spliceosome; embryonic development via the syncytial blastoderm; response to stress; neurotransmitter secretion; nervous system development; axon guidance; axonal fasciculation; vesicle-mediated transport; RNA interference.<br>**Celular component:** nucleus; cytoplasm; lipid particle; microtubule associated complex; perinuclear region of cytoplasm; precatalytic spliceosome; catalytic step 2 spliceosome; Z disc.
34	TR	LG3	27541368	27541686	**Transcriptin:** Part of transcript from gene "D6WG02 (Q9VUH4)".<br>**Molecular function:** transferase activity, transferring hexosyl groups; alpha-1,3-mannosylglycoprotein 4-beta-N-acetylglucosaminyltransferase activity*.<br>**Biological process:** carbohydrate metabolic process.<br>**Celular component:** membrane.
35	BA	LG8	1951867	1952561	**(left)** Distant approximately 386kb from gene "D6WYD1"<br>**Molecular function:** Rab GTPase activator activity.<br>**Biological process:** positive regulation of Rab GTPase activity.

Name	Variant	Location	Start	End	Genome View
					**Celular component:** intracellular.<br><br>**(right)** Distant approximately 59kb from gene "D6WYN3 (Q9VSC9)"<br>**Molecular function*:** chitinase activity; hydrolase activity, acting on glycosyl bonds.<br>**Biological process:** unknown.<br>**Celular component:** unknown.
36	B	LG8	896125	896320	**(right)** Distant 262bp from gene "D6WYA1"<br>**Molecular function:** nucleic acid binding; zinc ion binding.<br>**Biological process:** DNA integration.<br>**Celular component:** unknown.
37	TR	LG8	1528414	1529244	**Transcriptin:** Part of transcript from gene "D6WYC9".<br>**Molecular function:** unknown.<br>**Biological process:** lipopolysaccharide biosynthetic process.<br>**Celular component:** unknown.
38	TR	LG8	5308401	5308982	**Transcriptin:** Part of transcript from gene "D6WV42".<br>**Molecular function:** unknown.<br>**Biological process:** unknown.<br>**Celular component:** unknown.
39	TR	LG8	774647	775827	**(left)** Distant approximately 7kb from gene "D6WYA0 (Q94534)"<br>**Molecular function:** unknown.<br>**Biological process*:** axon guidance; defasciculation of motor neuron axon; axon choice point recognition; Bolwig's organ morphogenesis.<br>**Celular component:** membrane.
40	TR	LG8	3944782	3945221	**Transcriptin:** Part of transcript from gene "D6WUX6".<br>**Molecular function:** unknown.<br>**Biological process:** unknown.<br>**Celular component:** unknown.
41	ABA	LG7	2265910	2266834	**Transcriptin:** Part of transcript from gene "D6X0E1 (Q9VFD9)".<br>**Molecular function:** unknown.<br>**Biological process*:** behavioral response to ethanol.<br>**Celular component:** unknown.
42	TR	LG7	12042178	12043082	**(left)** Distant approximately 17kb from gene "D6WPX8 (Q9VCX4)"<br>**Molecular function:** nucleotide binding; GTP binding; GTPase activity.<br>**Biological process:** GTP catabolic process; mitochondrial translation*; ribosome disassembly*.

Name	Variant	Location	Start	End	Genome View
					**Celular component*:** mitochondrion.<br><br>**(right)** Distant approximately 1,5kb from gene "A2AX72 (Q9VPT1)"<br>**Molecular function:** signal transducer activity; receptor activity; G-protein coupled receptor activity; taste receptor activity*.<br>**Biological process:** signal transduction; G-protein coupled receptor signaling pathway; sensory perception of taste; behavior*; detection of carbon dioxide*; sensory perception of smell*; response to carbon dioxide*; response to stimulus*; detection of chemical stimulus involved in sensory perception of taste*.<br>**Celular component:** membrane; integral to membrane; plasma membrane; dendrite*; neuronal cell body*.
43	AAAA	LG7	4818766	4820205	**Transcriptin:** Part of transcript from gene "D6WTD1 (Q9W2M7)".<br>**Molecular function:** catalytic activity; hydrolase activity, hydrolyzing O-glycosyl compounds; chitinase activity; hydrolase activity; hydrolase activity, acting on glycosyl bonds; cation binding.<br>**Biological process:** carbohydrate metabolic process; chitin metabolic process; chitin catabolic process; metabolic process.<br>**Celular component*:** extracellular region.
44	TR	LG7	9703400	9704213	**Transcriptin:** Part of transcript from gene "D6WPE6 (P17970)".<br>**Molecular function:** voltage-gated potassium channel activity.<br>**Biological process:** potassium ion transport; regulation of ion transmembrane transport*; potassium ion transmembrane transport*.<br>**Celular component:** membrane; voltage-gated potassium channel complex.
45	ABA	LG4	1384466	1385028	**(left)** Distant approximately 9kb from gene "D2A2C6 (Q9V3S3)"<br>**Molecular function:** DNA binding.<br>**Biological process*:** spermatid nucleus differentiation.<br>**Celular component*:** chromatin.<br><br>**(right)** Distant approximately 11kb from gene "D2A2D1"<br>**Molecular function:** ionotropic glutamate receptor activity; extracellular-glutamate-gated ion channel activity.<br>**Biological process:** unknown.<br>**Celular component:** membrane.
46	AB	LG4	2320883	2321483	**(left)** Distant approximately 6kb from gene "D2A2I0"<br>**Molecular function:** unknown.<br>**Biological process:** unknown.<br>**Celular component:** unknown.<br><br>**(right)** Distant approximately 7kb from gene "D2A2I1 (P48591)"<br>**Molecular function:** ribonucleoside-diphosphate reductase activity; ATP binding; oxidoreductase activity.

Name	Variant	Location	Start	End	Genome View
					**Biological process:** DNA replication; oxidation-reduction process; activation of caspase activity*; deoxyribonucleotide biosynthetic process*.<br>**Celular component:** ribonucleoside-diphosphate reductase complex.
47	TR	LG4	5029909	5030416	**Transcriptin:** Part of transcript from gene " D1ZZG6 (Q9VLW2)".<br>**Molecular function:** nucleotide binding; ATP binding; microtubule motor activity.<br>**Biological process:** microtubule-based movement.<br>**Celular component:** microtubule; cytoplasm*; cytoskeleton*.
48	TR	LG4	12821659	12822035	**Transcriptin:** Part of transcript from gene "D2A2P8".<br>**Molecular function:** unknown.<br>**Biological process:** unknown.<br>**Celular component:** unknown.
49	BA	LG2	934977	935572	**(left)** Distant approximately 60kb from gene "D6WB65 (P20105)"<br>**Molecular function:** sequence-specific DNA binding transcription factor activity.<br>**Biological process:** regulation of transcription, DNA-dependent; autophagy*; multicellular organismal development*; cell death*; salivary gland cell autophagic cell death*; regulation of development, heterochronic*; oogenesis*.<br>**Celular component:** nucleus.<br><br>**(right)** Distant approximately 2,6kb from gene "D6WB73"<br>**Molecular function:** transmembrane transporter activity.<br>**Biological process:** transmembrane transport.<br>**Celular component:** integral to membrane.
50	AgBgA?	LG2	1290094	1291458	**(right)** Distant approximately 4,8kb from gene "D6WBG8 (Q9VUV9)"<br>**Molecular function:** nucleotide binding; ATP binding; ATP-dependent helicase activity; hydrolase activity; nucleoside-triphosphatase activity.<br>**Biological process*:** mRNA processing; RNA splicing.<br>**Celular component*:** nucleus; spliceosomal complex.
51	TR	LG2	298503	299069	**(left)** Distant approximately 8kb from gene "D6WB14 (P22270)"<br>**Molecular function:** signal transducer activity; receptor activity; G-protein coupled receptor activity; octopamine receptor activity.<br>**Biological process:** signal transduction; G-protein coupled receptor signaling pathway; sensory perception of smell*.<br>**Celular component:** integral to membrane; plasma membrane*.<br><br>**(right)** Distant approximately 16kb from gene "D6WB15"<br>**Molecular function:** structural constituent of cuticle.

Name	Variant	Location	Start	End	Genome View
					**Biological process:** unknown.<br>**Celular component:** unknown.
52	TR	LG2	451626	452670	**Transcriptin:** Part of transcript from gene " D6WB29 (P30432)".<br>**Molecular function:** hydrolase activity; peptidase activity; serine-type peptidase activity; serine-type endopeptidase activity; ATP binding*; transmembrane receptor protein tyrosine kinase activity*.<br>**Biological process:** proteolysis; protein phosphorylation*; transmembrane receptor protein tyrosine kinase signaling pathway*.<br>**Celular component*:** membrane; integral to membrane.
53	TR	LG2	428375	429394	**(left)** Distant approximately 14kb from gene " A8DIV5"<br>**Molecular function:** receptor activity; ion channel activity; acetylcholine-activated cation-selective channel activity; extracellular ligand-gated ion channel activity.<br>**Biological process:** transport; ion transport.<br>**Celular component:** membrane; plasma membrane; integral to membrane; synapse; cell junction; postsynaptic membrane.<br><br>**(right)** Distant approximately 5kb from gene "D6WB29 (P30432)"<br>**Molecular function:** hydrolase activity; peptidase activity; serine-type peptidase activity; serine-type endopeptidase activity; ATP binding*; transmembrane receptor protein tyrosine kinase activity*.<br>**Biological process:** proteolysis; protein phosphorylation*; transmembrane receptor protein tyrosine kinase signaling pathway*.<br>**Celular component*:** membrane; integral to membrane.
54	ABA	LG10	1397507	1398376	**(left)** Distant approximately 10kb from gene "D6X3I9 (Q05913)"<br>**Molecular function:** DNA binding; catalytic activity; transferase activity*.<br>**Biological process:** transcription initiation from RNA polymerase II promoter; positive regulation of transcription, DNA-dependent.<br>**Celular component:** nucleus.<br><br>**(right)** Distant approximately 6,6kb from gene "D6X3J1"<br>**Molecular function:** unknown.<br>**Biological process:** unknown.<br>**Celular component:** unknown.
55	BAB	LG10	9243139	9243842	**Transcriptin:** Part of transcript from gene " D6X4P3 (Q7KMM4)".<br>**Molecular function:** catalytic activity; hydrolase activity, hydrolyzing O-glycosyl compounds; carbohydrate binding; alpha-glucosidase activity*; maltose alpha-glucosidase activity*.<br>**Biological process:** carbohydrate metabolic process.

Name	Variant	Location	Start	End	Genome View
					**Celular component*:** microtubule associated complex.
56	B	LG10	995570	995824	**(left)** Distant approximately 2,2kb from gene "D6X3H5 (Q94887)"<br>**Molecular function:** unknown.<br>**Biological process:** cell adhesion; establishment or maintenance of cell polarity*; dorsal closure*; protein localization*; axon ensheathment*; synaptic vesicle targeting*; synaptic vesicle docking involved in exocytosis*; septate junction assembly*; nerve maturation*; regulation of tube size, open tracheal system*; cell-cell junction organization*; establishment of glial blood-brain barrier*; terminal button organization*; presynaptic membrane assembly*.<br>**Celular component:** membrane; integral to membrane; integral to plasma membrane*; septate junction*; pleated septate junction*; cell junction*; presynaptic active zone*.<br><br>**(right)** Distant approximately 15kb from gene "D6X3H7 (Q9VBP6)"<br>**Molecular function:** oxidoreductase activity, acting on the aldehyde or oxo group of donors, NAD or NADP as acceptor; succinate-semialdehyde dehydrogenase activity*.<br>**Biological process:** metabolic process; oxidation-reduction process.<br>**Celular component:** unknown.
57TR	TR	LG10	10249341	10250103	**Transcriptin:** Part of transcript from gene "D6X4V6".<br>**Molecular function:** unknown.<br>**Biological process:** intracellular signal transduction.<br>**Celular component:** intracellular.
58	BA	LG10	1554285	1554971	**(left)** Distant approximately 1kb from gene "D6X3J6 (Q9VEJ9)"<br>**Molecular function:** peroxidase activity; heme binding; oxidoreductase activity*.<br>**Biological process:** oxidation-reduction process; response to oxidative stress.<br>**Celular component:** unknown.<br><br>**(right)** Distant approximately 26,6kb from gene "D6X3J7 (Q9VHI1)"<br>**Molecular function*:** transcription factor binding.<br>**Biological process*:** compound eye morphogenesis; imaginal disc-derived leg morphogenesis; chaeta morphogenesis; imaginal disc-derived wing vein morphogenesis; imaginal disc-derived wing margin morphogenesis; Wnt receptor signaling pathway; positive regulation of smoothened signaling pathway; positive regulation of transcription, DNA-dependent.<br>**Celular component*:** protein complex; Cdc73/Paf1 complex.
59	BA?	LG6	6976682	6977189	**Transcriptin:** Part of transcript from gene "D2A693 (Q9V6L0)".<br>**Molecular function:** DNA binding; oxidoreductase activity, acting on single donors with incorporation of molecular oxygen, incorporation of two atoms of oxygen*; metal ion binding*; histone demethylase activity (H3-K9 specific)*; histone demethylase activity (H3-K36 specific)*.<br>**Biological process*:** transcription, DNA-dependent; regulation of transcription, DNA-dependent; chromatin modification;

Name	Variant	Location	Start	End	Genome View
					histone demethylation; histone H3-K9 demethylation; negative regulation of transcription, DNA-dependent; oxidation-reduction process; histone H3-K36 demethylation.<br>**Celular component*:** nucleus.
60	TR	LG6	11779417	11780163	**Transcriptin:** Part of transcript from gene "D2A490 (Q8MKK4)".<br>**Molecular function:** substrate-specific transmembrane transporter activity; trehalose transmembrane transporter activity*.<br>**Biological process:** transport; transmembrane transport; trehalose transport*.<br>**Celular component:** membrane; integral to membrane; plasma membrane*; plasma membrane part*.
61	TR	LG6	8735958	8736740	**(left)** Distant approximately 116kb from gene "D2A6I4"<br>**Molecular function:** ion channel activity; potassium channel activity.<br>**Biological process:** potassium ion transmembrane transport.<br>**Celular component:** membrane; integral to membrane.<br><br>**(right)** Distant approximately 4,8kb from gene "D2A6I6"<br>**Molecular function:** transporter activity.<br>**Biological process:** transport.<br>**Celular component:** intracellular.
62	AB	LG6	9904860	9905156	**(left)** Distant approximately 37kb from gene "D2A3V0 (P15278)"<br>**Molecular function:** unknown.<br>**Biological process*:** cell adhesion; homophilic cell adhesion; multicellular organismal development; nervous system development; axon guidance; axonal fasciculation; learning or memory; synaptic target recognition; olfactory learning; synaptic target attraction; cell differentiation; ovarian follicle cell development.<br>**Celular component:** membrane; basolateral plasma membrane*; integral to membrane*; lateral plasma membrane*; plasma membrane*; septate junction*.<br><br>**(right)** Distant approximately 22kb from gene "D2A3V3 (Q8IR79)"<br>**Molecular function:** ATP binding; metal ion binding; nucleotide binding; protein kinase activity; transferase activity, transferring phosphorus-containing groups; zinc ion binding; kinase activity*; protein kinase binding*; protein serine/threonine kinase activity*.<br>**Biological process:** protein phosphorylation; actin cytoskeleton organization*; compound eye development*; establishment of imaginal disc-derived wing hair orientation*; establishment of planar polarity*; imaginal disc morphogenesis*; phosphorylation*; regulation of axonogenesis*; synapse assembly*; synaptic growth at neuromuscular junction.<br>**Celular component*:** actomyosin contractile ring; cell cortex; cleavage furrow; cytoplasm; midbody.
63	BA	LGX	10766673	10767192	**Transcriptin:** Part of transcript from gene "D6W8F4".

Name	Variant	Location	Start	End	Genome View
					**Molecular function:** zinc ion binding.<br>**Biological process:** unknown.<br>**Celular component:** intracellular.
64	TR	LGX	10594562	10595409	**(left)** Distant approximately 2kb from gene "D6W8D3 (O96681)"<br>**Molecular function:** receptor activity; protein binding*; semaphorin receptor activity*.<br>**Biological process:** signal transduction; multicellular organismal development; axon guidance*; axon midline choice point recognition*; motor axon guidance*; semaphorin-plexin signaling pathway involved in regulation of photoreceptor cell axon guidance*.<br>**Celular component:** intracellular; membrane; integral to membrane.<br><br>**(right)** Distant approximately 6,5kb from gene "D6WGD2 (Q9VRU1)"<br>**Molecular function:** catalytic activity; isomerase activity; carbohydrate binding; aldose 1-epimerase activity*.<br>**Biological process:** carbohydrate metabolic process; hexose metabolic process.<br>**Celular component:** unknown.
65	B	LG5	15212039	15212315	**Transcriptin:** Part of transcript from gene "B3MMG1".<br>**Molecular function:** calcium ion binding.<br>**Biological process:** cell adhesion; homophilic cell adhesion.<br>**Celular component:** membrane; plasma membrane; integral to membrane.
66	TR	LG5	3545168	3546061	**(left)** Distant approximately 2,5kb from gene "D6WNN6 (Q9VJJ7)"<br>**Molecular function:** ion channel activity; calcium channel activity; cation channel activity*; protein binding*.<br>**Biological process:** calcium ion transmembrane transport; visual perception*; response to light stimulus*; detection of light stimulus involved in visual perception*.<br>**Celular component:** membrane; integral to membrane; rhabdomere*.<br><br>**(right)** Distant approximately 27kb from gene "A3RE80 (Q868T3)"<br>**Molecular function:** G-protein coupled receptor activity; receptor activity; signal transducer activity; vasopressin receptor activity; peptide receptor activity*.<br>**Biological process:** signal transduction; G-protein coupled receptor signaling pathway; ecdysis, chitin-based cuticle*.<br>**Celular component:** integral to membrane; integral to plasma membrane*.
67	A?B	LG5	5611994	5612488	**Transcriptin:** Part of transcript from gene "A1JUG2 (P20153)".<br>**Molecular function:** DNA binding; ligand-dependent nuclear receptor activity; metal ion binding; receptor activity; sequence-specific DNA binding; sequence-specific DNA binding transcription factor activity; steroid binding; steroid hormone receptor activity; zinc ion binding; ecdysteroid hormone receptor activity*; juvenile hormone binding*; lipid binding*; protein binding*; protein heterodimerization activity*; protein homodimerization activity*.<br>**Biological process:** intracellular receptor mediated signaling pathway; regulation of transcription, DNA-dependent; steroid

Name	Variant	Location	Start	End	Genome View
					hormone mediated signaling pathway; transcription, DNA-dependent; border follicle cell migration*; dendrite morphogenesis*; ecdysone receptor-mediated signaling pathway*; ecdysone-mediated induction of salivary gland cell autophagic cell death*; germ cell development*; muscle organ development*; negative regulation of cell differentiation*; negative regulation of transcription, DNA-dependent*; neuron development*; neuron remodeling*; positive regulation of transcription, DNA-dependent*; regulation of development, heterochronic*.<br>**Celular component:** nucleus; polytene chromosome*; ecdysone receptor holocomplex*; dendrite*.
68	A	LG5	3048045	3048267	**(left)** Distant approximately 15kb from gene "D6WNB3 (O46173)"<br>**Molecular function:** nucleic acid binding; DNA binding; mRNA binding*.<br>**Biological process:** regulation of transcription, DNA-dependent; nuclear mRNA splicing, via spliceosome*; oogenesis*.<br>**Celular component*:** precatalytic spliceosome; catalytic step 2 spliceosome.<br><br>**(right)** Distant approximately 350kb from gene "D6WNB6"<br>**Molecular function:** translation release factor activity, codon specific.<br>**Biological process:** translational termination.<br>**Celular component:** cytoplasm.

Composition of dispersed TCAST-like elements (A: Tcast1a, B: Tcast1b, ?: Tcast1a or Tcast1b, g: sequence gap between A and B, TR: transposon), their chromosomal location and start and end sites. Detailed description of neighbouring genes including molecular function of their protein products, biological processes in which these proteins are involved and their cellular localization (cellular component) is shown.
