# Supplementary material for: Satellite DNA-Like Elements Associated With Genes Within Euchromatin of the Beetle Tribolium castaneum
Source: G3 (Bethesda). 2012 Aug 1;2(8):931–41. doi: 10.1534/g3.112.003467 (PMC3411249; doi:10.1534/g3.112.003467)
Supplement: Supporting Information [file supp_2.8.931_FigureS1.pdf]

TCAST-like elements

Chromosome	1	2	3	4	5	6	7	8	9	10	
RawP_value	ns	ns	ns	ns	ns	ns	ns	ns	ns	ns	total
Sat_elements	2	5	17	4	4	4	4	6	17	5	68

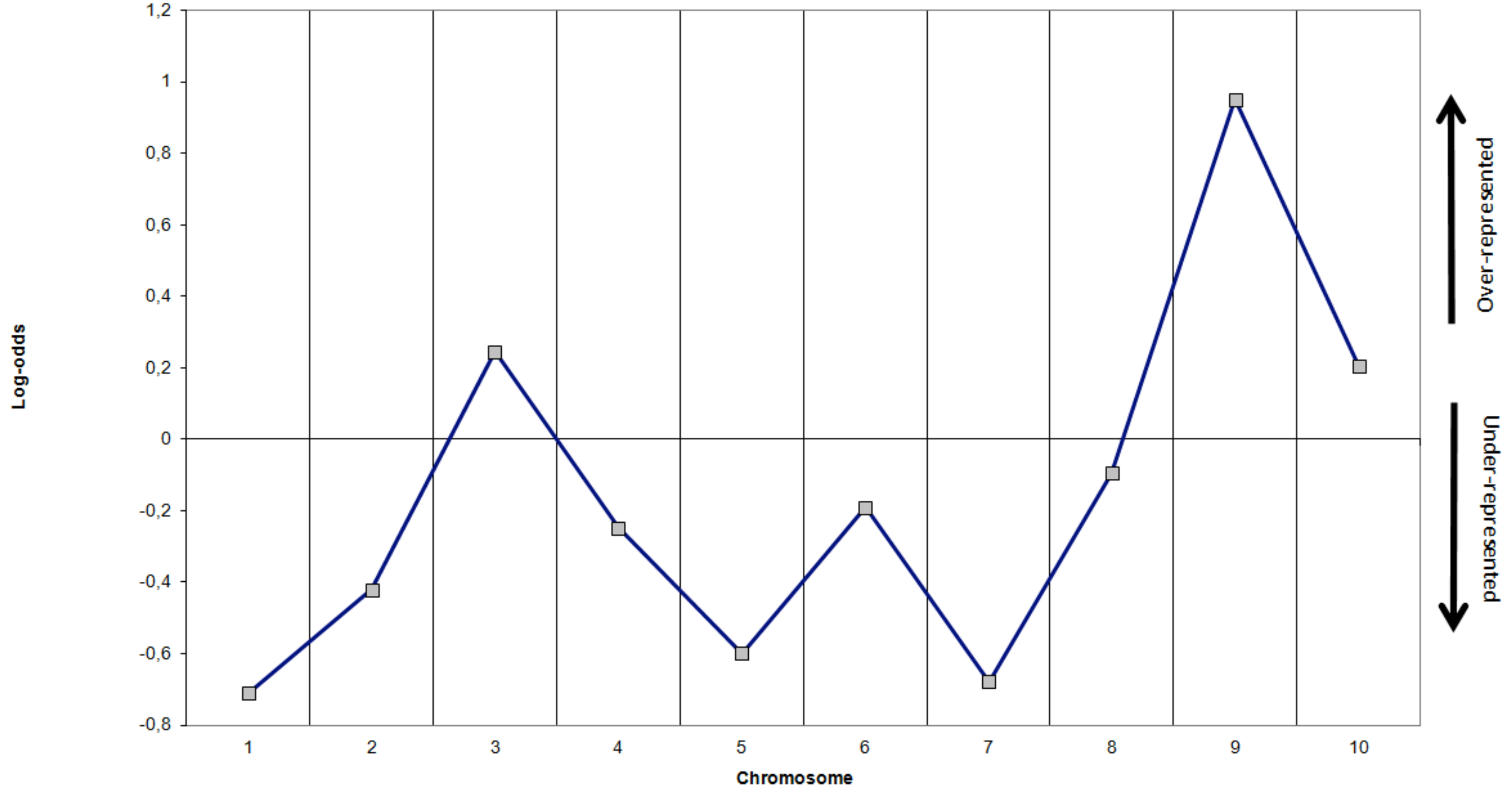

Figure S1 In each chromosome the frequency of TCAST-like elements is compared with the frequency in the complete sample and deviations are shown by log-odds (y-axis). Log-odds of zero denotes that the frequency of TCAST-like elements in chromosome and in the complete sample do not differ, whereas positive and negative values point to over-representation and under-representation, respectively. Significance of the deviations is shown in the p-value chart..
