# Supplementary material for: Satellite DNA-Like Elements Associated With Genes Within Euchromatin of the Beetle Tribolium castaneum
Source: G3 (Bethesda). 2012 Aug 1;2(8):931–41. doi: 10.1534/g3.112.003467 (PMC3411249; doi:10.1534/g3.112.003467)
Supplement: Supporting Information [file supp_2.8.931_FigureS3.pdf]

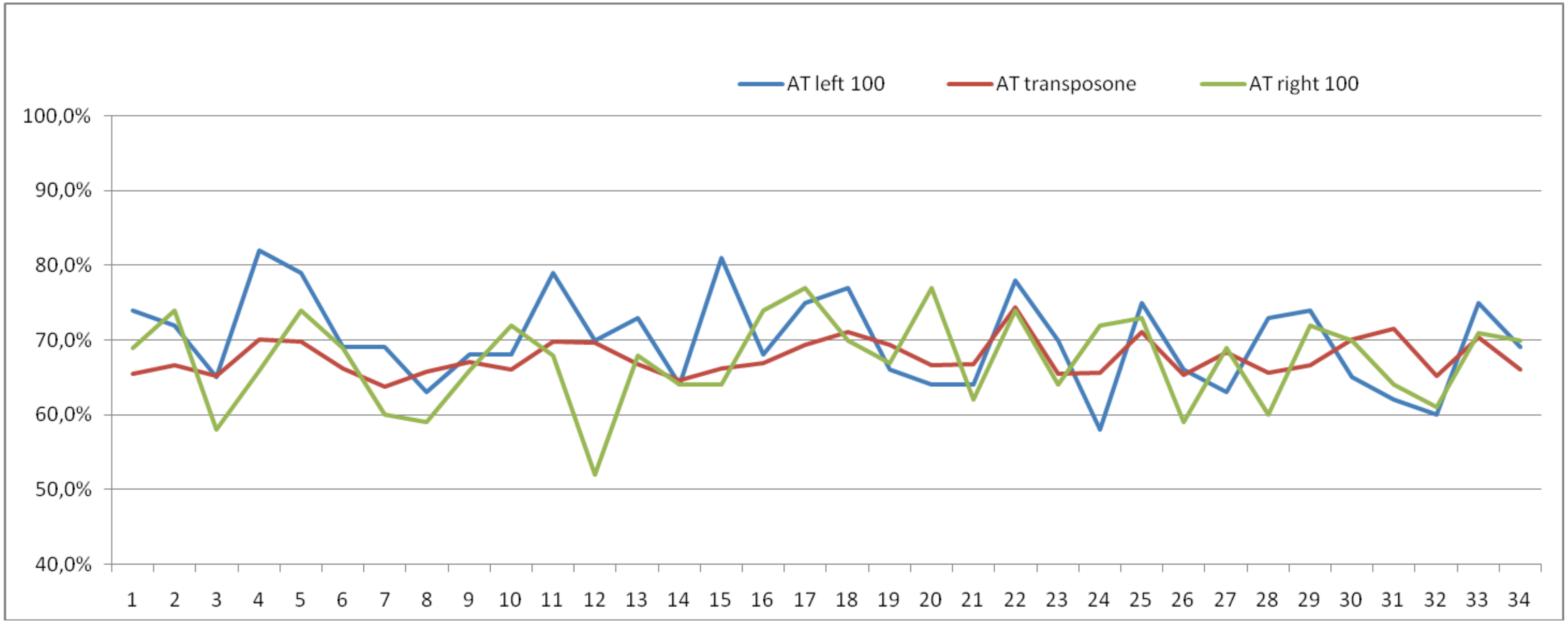

Figure S3 AT content within 100 bp of the flanking regions for each of TCAST satellite-like elements, both from 5' (blue) and 3' site (green), and from each TCAST transposone-like element (red).
