# Supplementary figures and images for: Satellite DNA-Like Elements Associated With Genes Within Euchromatin of the Beetle Tribolium castaneum
Source: G3 (Bethesda). 2012 Aug 1;2(8):931–41. doi: 10.1534/g3.112.003467 (PMC3411249; doi:10.1534/g3.112.003467)

## Slide 1
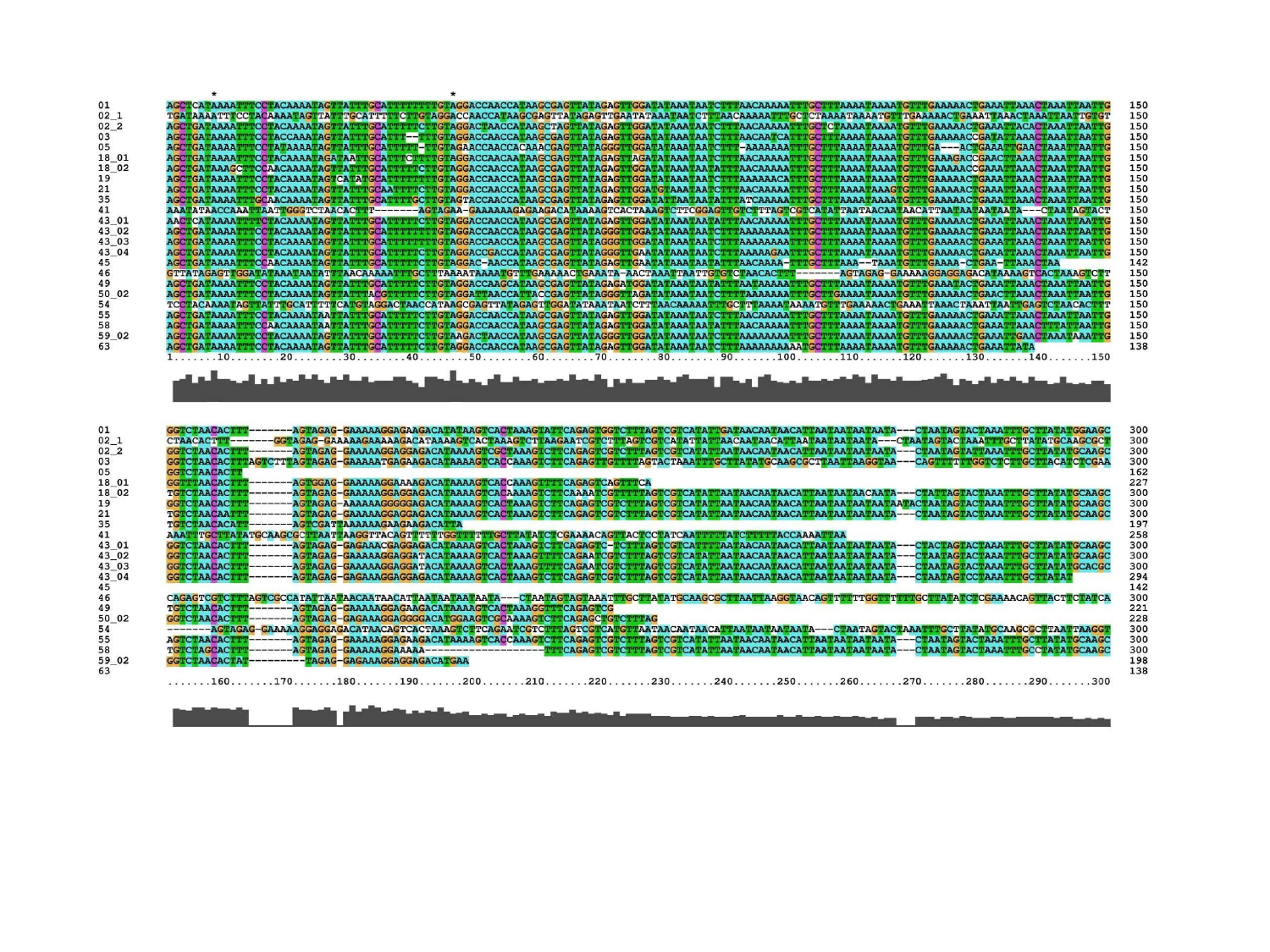

## Slide 2
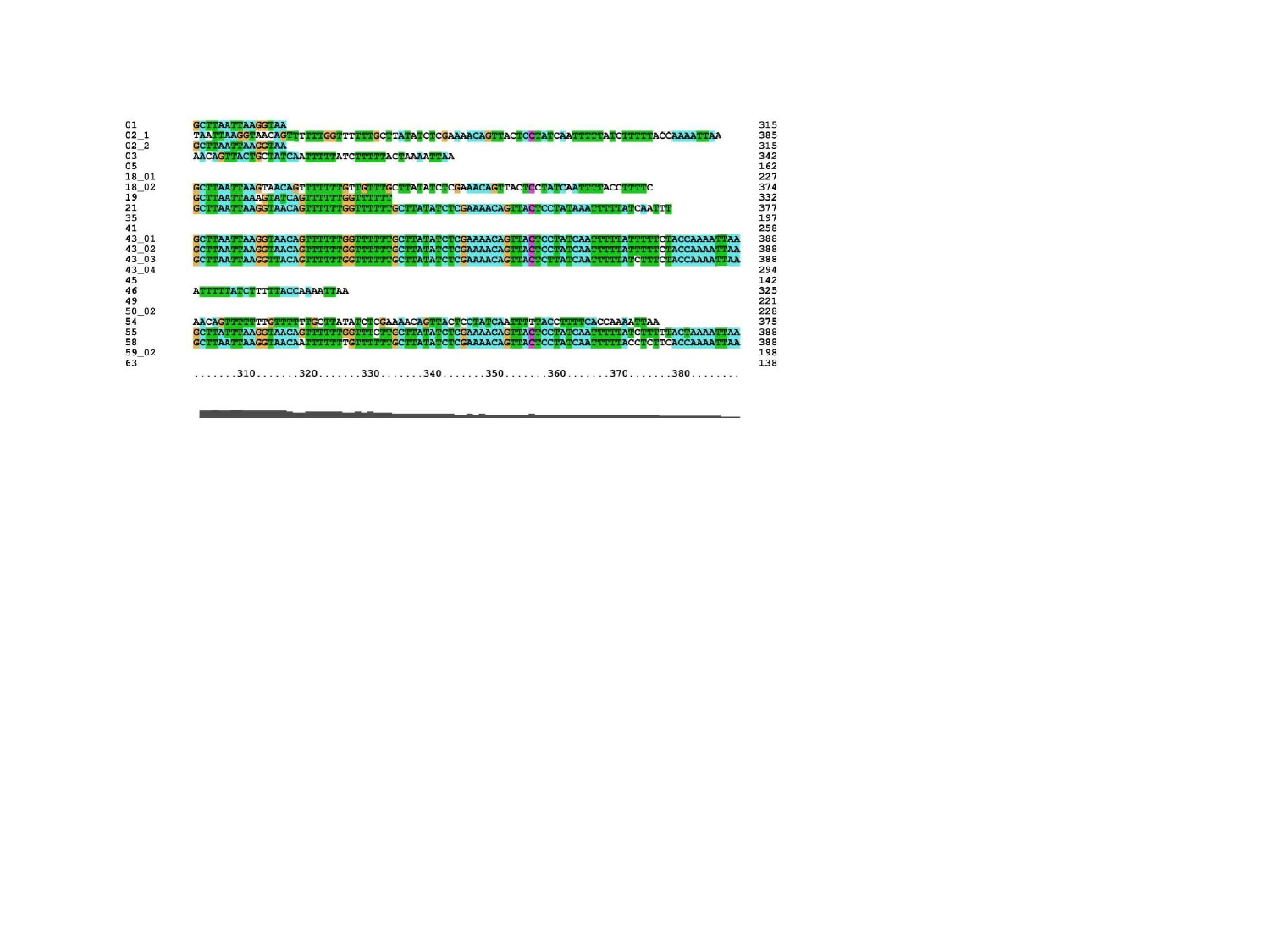

Supplement: Supporting Information [file supp_2.8.931_FileS1.ppt]

## Slide 1
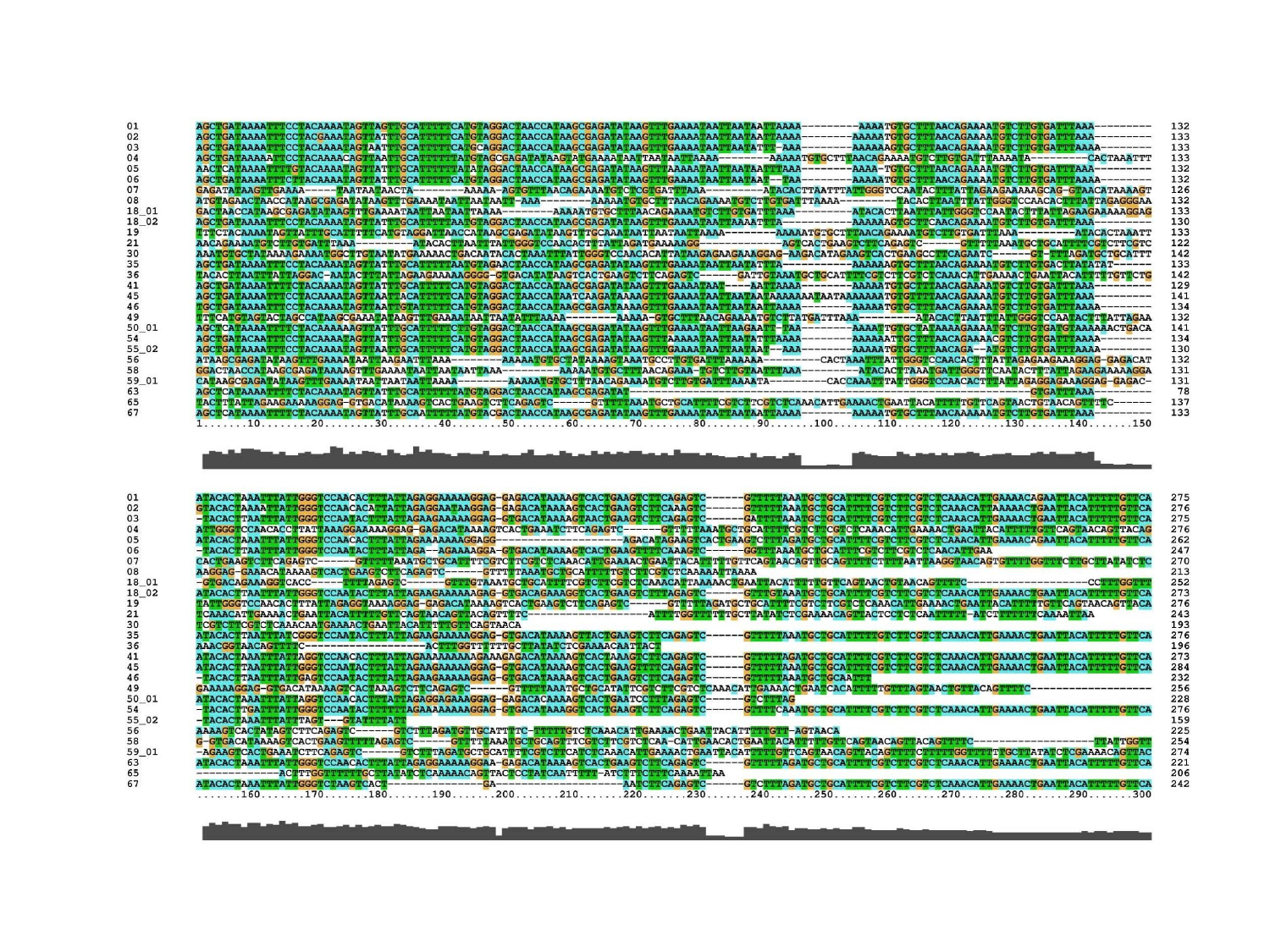

## Slide 2
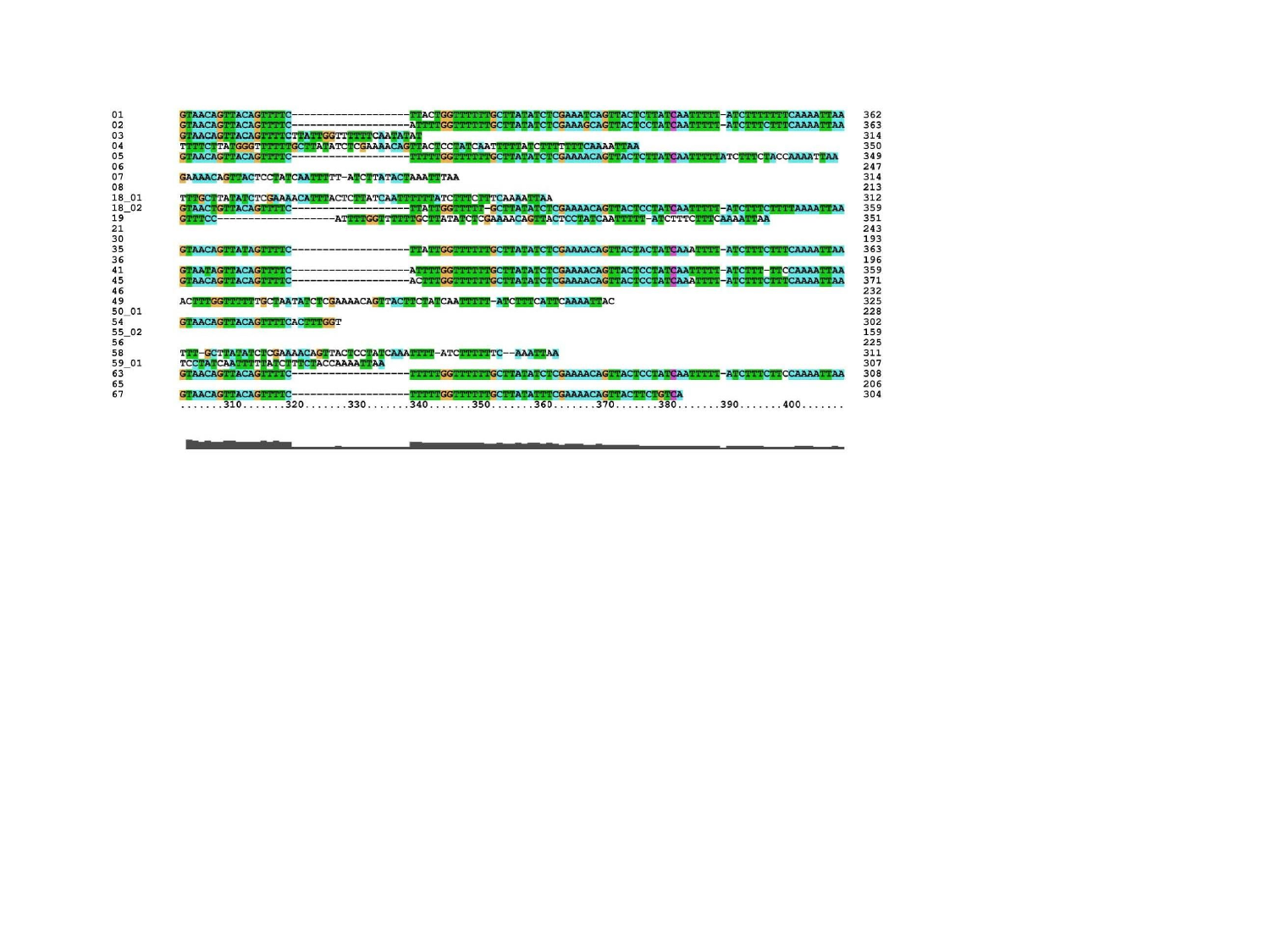

Supplement: Supporting Information [file supp_2.8.931_FileS2.ppt]

## Slide 1
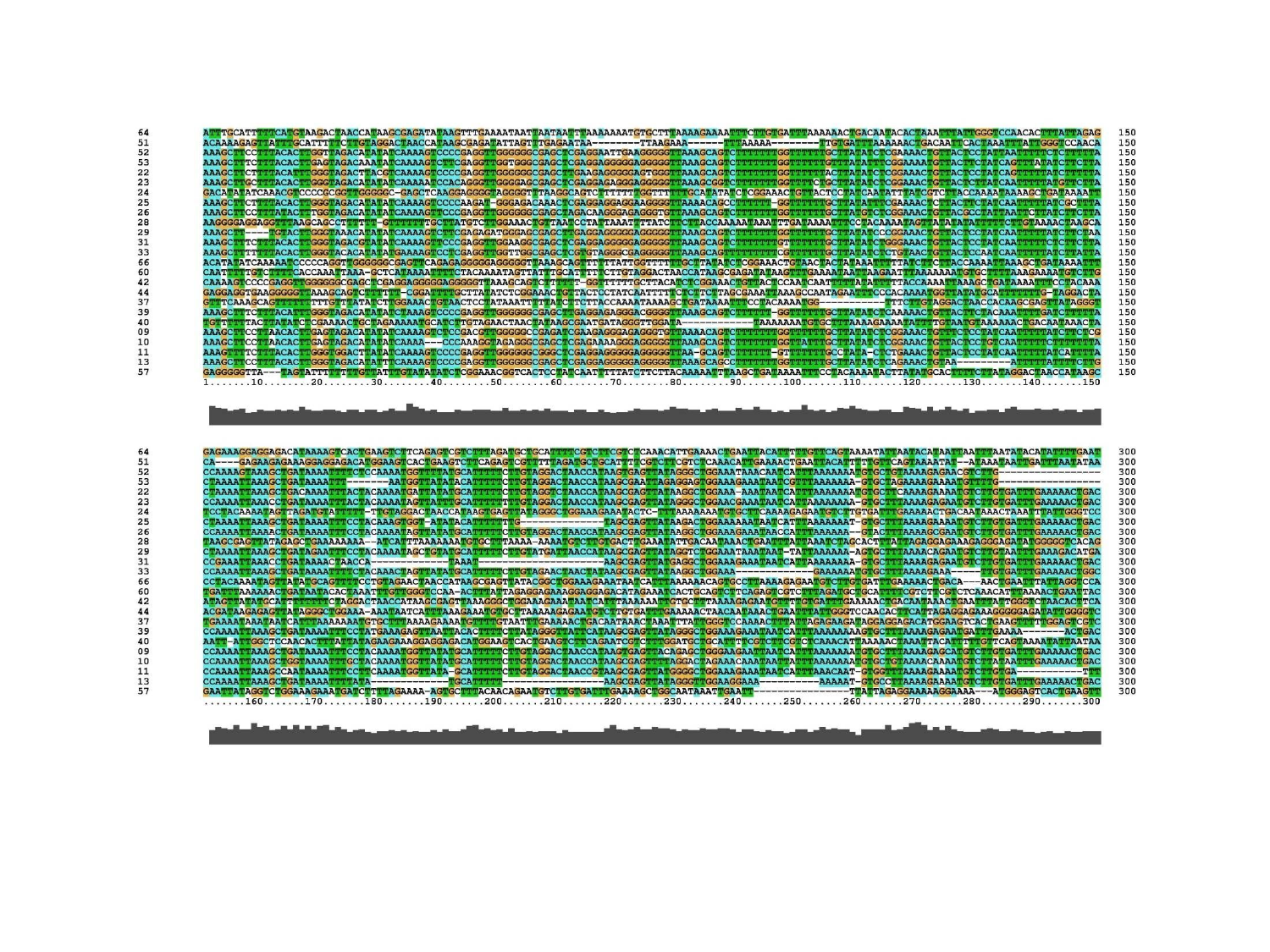

## Slide 2
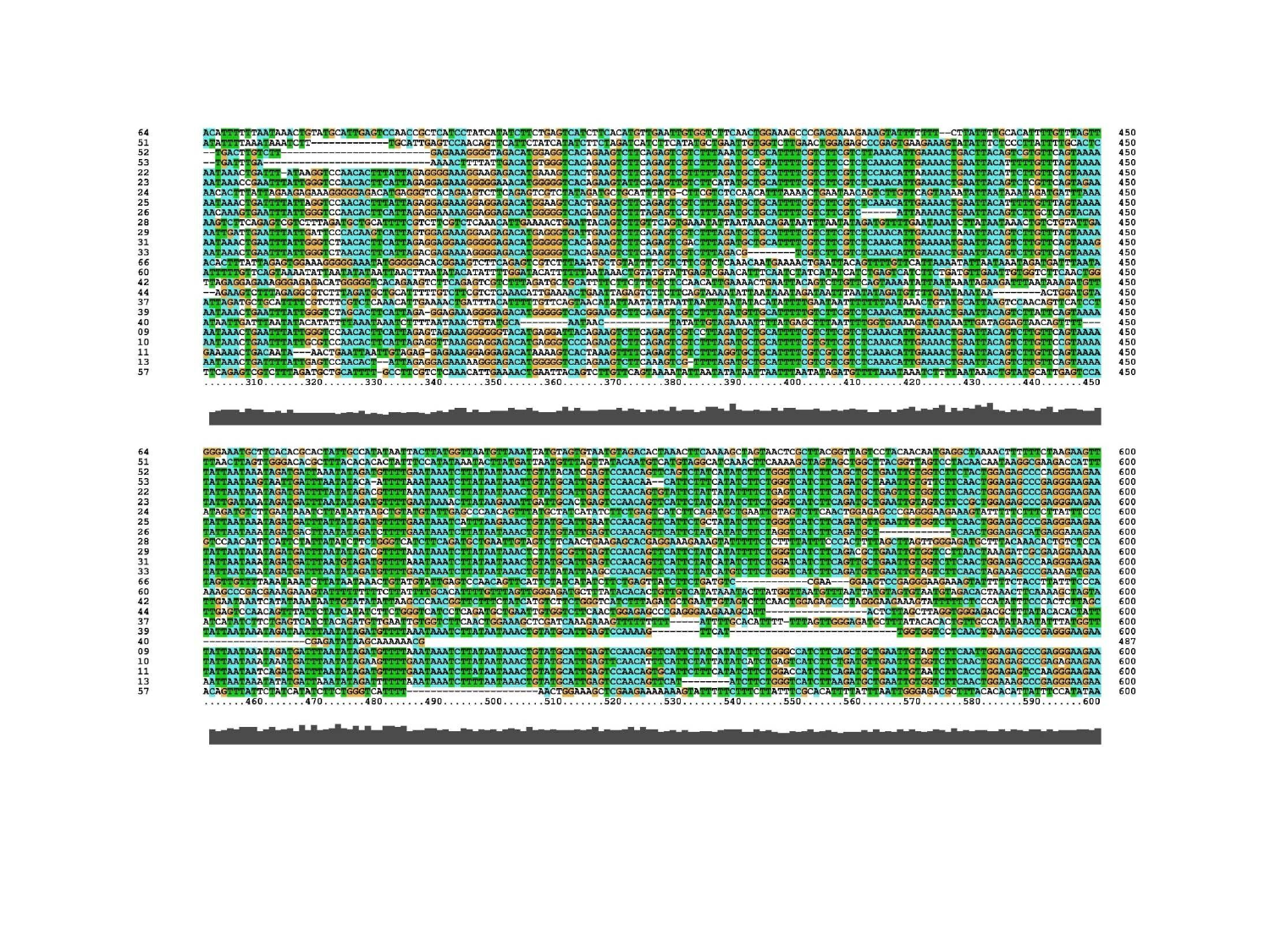

## Slide 3
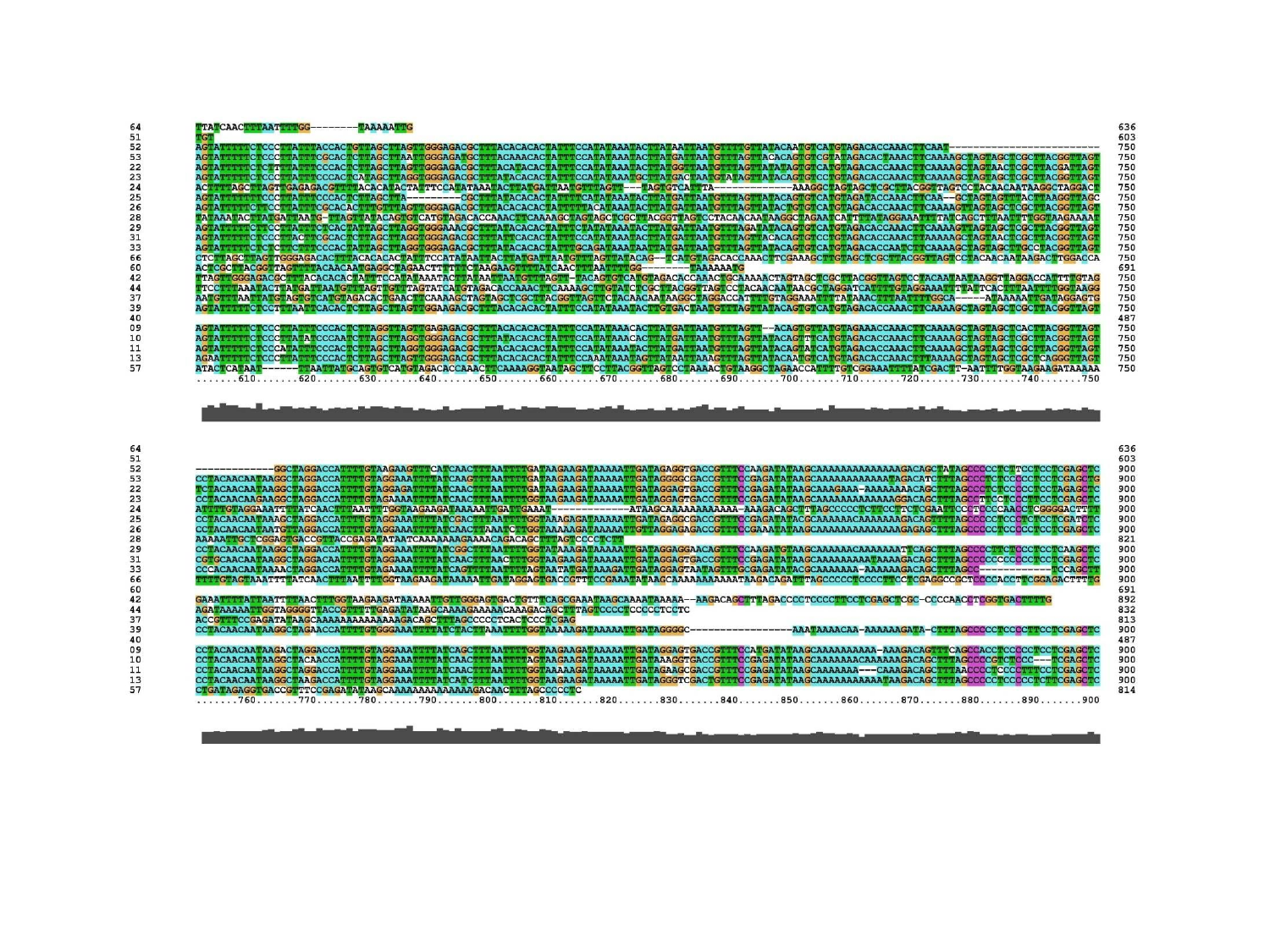

## Slide 4
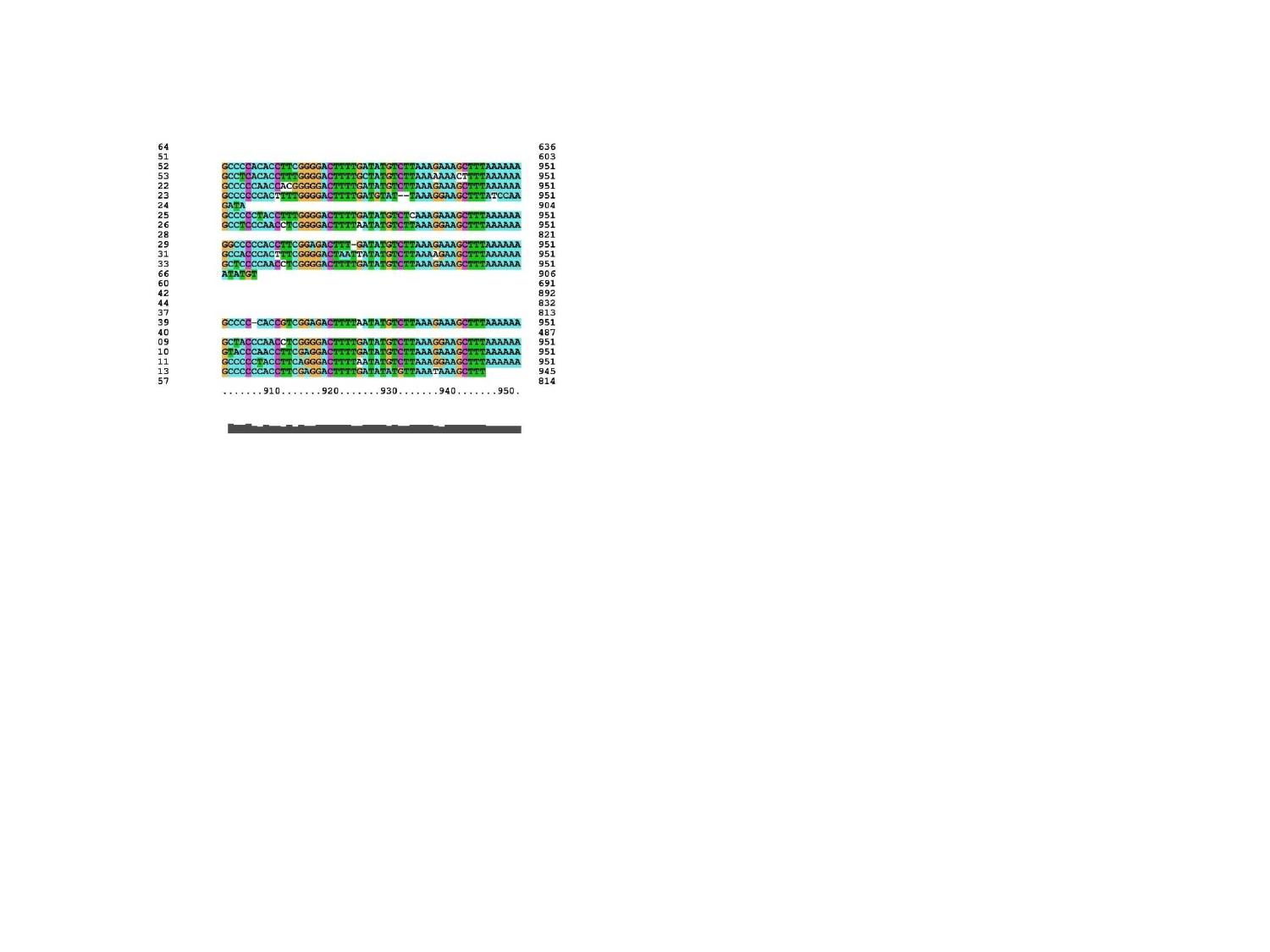

Supplement: Supporting Information [file supp_2.8.931_FileS3.ppt]
